# Supplementary material for: Effects of a locally available dietary interventions counselling on the community-based management of anaemia in children under five years in Ghana: Kumbungu cluster randomized controlled trial protocol
Source: PLoS One. 2022 Apr 21;17(4):e0266157. doi: 10.1371/journal.pone.0266157 (PMC9022816; doi:10.1371/journal.pone.0266157)
Supplement: S1 Text — (DOCX) [file pone.0266157.s001.docx]

**West African College of Physicians**

**Faculty of Community Health**

**Part II proposal**

**Effects of counselling on Locally Available Dietary Intervention, in the community-based management of anaemia among children under five in Kumbungu, Northern region, Ghana: Kumbungu Cluster Randomized Controlled Trial**

**Benjamin D. Nuertey**

**April 2019**

**ABSTRACT**

**Background:** Anaemia in children under five years is an important public health problem requiring urgent attention. The global prevalence is worse in Africa and Asia. In Ghana seven out of ten children under five years are anaemic. Anaemia is associated with significant morbidity and mortality affecting physical and mental development of children. The aim of the study is to evaluate the safety and efficacy of Locally Available Dietary Intervention in combination with Iron Folic Acid in the community based management of anaemia in children under five years.

**Method:** The study would be a Community-based cluster randomized, controlled trial, with two parallel arms. Cluster assignment is at the level of household for the locally available dietary (LAD) intervention. All eligible children within the household would be included in the study. All study participants would receive iron and folic acid daily for three months. The study would follow the closed cohort of eligible households with children aged between 6 to 56 months at baseline for 12 weeks. Primary outcome data would be collected at baseline (pre-intervention) and then at the end of 12 weeks (post intervention). Eligible children within the household are the unit for analysis. The two arms of the study are; Iron + Folic Acid (IFA) arm and Iron + Folic Acid + Locally available dietary intervention (IFA+LAD arm). Outcome measures mainly pertain to individual participant level. The study will evaluate the effect of Locally Available Dietary interventions on the prevalence of anaemia in the study arm. The primary outcome measures are mean haemoglobin levels and prevalence of anaemia in study arms. A two-sample Student’s t-test assuming equal variances would be performed to test the hypothesis that the resulting mean haemoglobin from the two arms of the study are equal.

**Table of Contents**

Title pagei

Abstractii

Table of Contentsiii

Table of figuresvi

List of Tablesvii

Abbreviationsviii

**Chapter one Introduction1**

1.1 Background1

1.2 Statement of the Problem3

1.3 Justification5

1.4 Hypothesis6

1.5 Research Questions6

1.6 Aims and Objectives7

1.6.1 Aim7

1.6.2 Specific Objectives7

1.7 Conceptual framework7

**Chapter Two- Literature Review9**

2.0 Introduction9

2.1 Classification of anaemias in children under five9

2.2 Anaemia as a public Health Problem in children under five10

2.2.1 Prevalence, Burden and Distribution10

2.2.2 Aetiologies of anaemia in children under five11

2.2.3 Determinants of anaemia in children under five12

2.2.3.1 Biological determinants 12

2.2.3.2 Genetic determinants13

2.2.3.3 Infectious and inflammation determinants14

2.2.3.4 Socioeconomic and Environmental determinants15

2.3 Consequence of Anaemia15

2.3.1 Medical consequence16

2.3.2 Child neuro-developmental consequence16

2.3.3 Economic consequence17

2.4 Clinical presentation18

2.5 Risk factors 18

2.6 Diagnosis of anaemia19

2.7 Inhibitors and enhancers of iron absorption 19

2.8 Interventional strategies to address anaemia in children under five21

2.9 Deworming as an adjunct to iron and folic acid in the management of anaemia21

2.10 Outstanding research questions22

**Chapter three - Methodology23**

3.1 Study Site23

3.2 Study Design25

3.2.1 Study arms26

3.3 Interventions26

3.4 Description of Study Population27

3.5 Inclusion and Exclusion criteria27

3.5.1 Inclusion criteria27

3.5.1.1 Inclusion at cluster level27

3.5.1.2 Inclusion at the participant level28

3.5.2 Exclusion criteria28

3.6 Definition of variables28

3.7 Sample size calculations29

3.8 Sampling process30

3.8.1 First stage: community selection30

3.8.2 Second stage: Household and individual selection31

3.9 Recruitment of study participants31

3.10 Randomization: Sequence generation, stratification and blocking31

3.11 Allocation concealment mechanism and blinding32

3.12 Outcome measures33

3.12.1 Primary Outcome33

3.12.2 Secondary Outcome33

3.12.2.1 Anthropometry; physical growth33

3.12.2.2 Biochemical outcome measures 35

3.12.2.3 Clinical assessment35

3.12.2.4 Dietary recall and quantification of dietary content of micronutrients35

3.12.2.8 Household food insecurity access scale 37

3.12.2.9 Household dietary diversity score38

3.13 Procedure for data collection40

3.14 Project management41

3.14.1 Reconnaissance visit41

3.14.2 Development of field management plan, training plans and manuals41

3.14.3 Recruitment of interviewers42

3.14.4 Training of field staff42

3.14.5 Pre-testing of tools and skills43

3.14.6 Mobilisation of logistics and equipment43

3.14.7 Finalisation of tools and upload on survey platform43

3.15 Description of study tools44

3.15.1 Electronic data collection tool44

3.15.2 Questionnaires45

3.16 Development of Locally available dietary intervention45

3.17 Ethical consideration46

3.18 Data Collection and monitoring of field work47

3.19 Data cleaning and editing48

3.20 Data analysis49

3.21 Participant flow50

3.22 Work plan52

3.23 Budget53

**Reference56**

**Appendices62**

**TABLE OF FIGURES**

Figure 1.1: Conceptual framework8

Figure 3.1: Map showing Kumbungu district24

Figure 3.2 Consolidated standards of reporting trials (CONSORT) flow diagram for this study 51

**LIST OF TABLES**

Table 2.1: signs and symptoms of anaemia18

Table 3.1: Baseline and outcome measures, intermediary factors and time points38

Table 3.2 WHO guide for food diversification and enhancing bioavailability of absorbed micronutrients46

Table 3.3: Work plan52

Table 3.4 Budget53

**ABBREVIATIONS**

BCC Behavioural Change Communication

CHr Reticulocyte haemoglobin content,

CogCS Cognitive composite score

CONSORT consolidated standards of reporting trials

Hb Haemoglobin

HDDS Household dietary diversity score

HFIAS Household food insecurity access scale

FANTA Food and Nutrition Technical Assistance

IFA Iron + Folic Acid

MCHC Mean Corpuscular Haemoglobin Concentration

MCV Mean corpuscular volume

MUAC Mid Upper Arm circumference

NGOs Non-Governmental Organizations

LAD Locally available dietary

RR Risk Ratios

SSF subscapular skin fold

sTfR serum transferrin receptor

sTfR-F index sTfR/log ferritin

TSF Triceps skin fold thickness

USAID United States Agency for International Development

WHO World Health Organisation

**CHAPTER ONE – INTRODUCTION**

**1.1 Background**

Anaemia in children under five years is an important public health problem requiring urgent attention. Globally, 27% of the world’s population is anaemic of which developing countries account for more than 89%[1, 2]. Anaemia is among the top five causes of years lived with disability in the year 2016 [1]. Preschool children and pregnant women are the most affected. Globally, the prevalence of anaemia (defined as haemoglobin level of <110 g/L) in children aged 6–59 months is 43% [3]. The global prevalence is worse in Africa and Asia . In Africa, the prevalence of anaemia among children under the age of five is estimated at 62% [4]. This is above the 40% cut off limit of the World Health Organization’s (WHO) classification of anaemia as a severe public health problem [5]. The prevalence of anaemia in children under 5 is estimated at 71% for west Africa [6]. In Ghana, the overall prevalence of anaemia in children under five is 78.4% [7]. There are significant regional and community variations. In the northern region of Ghana, almost 9 out of 10 children under five years have anaemia [7]. Anaemia which is highly prevalent in the northern region of Ghana is also associated with significant effects on the lives of children.

Anaemia significantly contribute to under five morbidity and mortality in Africa. It has the potential of maintaining the cycle of poverty as it prevents children from attaining their full development potential [8]. These effects are often irreversible even if anaemia is corrected later in life [9]. In the shorterm, anaemia in children under five impairs cognitive, emotional, physical and brain development [5]. In the medium term, it negatively impacts on educational attainment and in the long-term, impacts negatively on adult life and earning abilities thereby increasing the likelihood of having anaemic children and maintaining the cycles of poverty and anaemia.

Mortality estimates showed that, 5.8 million children lower than five years died in 2015 [10]. Sub-Saharan Africa in 2013 contributed 25% of global births and 50% of global deaths of children under five years which is respectively projected to reach 33% births and 60% deaths by 2030 [11]. Anaemia contributes significantly to deaths within this age group either directly or indirectly by complicating other conditions such as malaria, malnutrition, diarhoea and pneumonia. It has been estimated that for each 1g/dl increase in hemoglobin, the risk of death falls by 24% [12]. Also 1.8 million deaths in children under five within Africa could be avoided by increasing the hemoglobin of these children by 1g/dl.

There is an urgent need to reduce the burden of anaemia in communities within developing countries. This has the potential of improving lives and averting many preventable deaths in children under-five years. Most of the causes of anaemia in children under-five are preventable. Globally, Iron deficiency anaemia is the dominant causes of anaemia accounting for more than 60% of all anaemia [13]. Iron deficiency anaemia in children within developing countries is partly due to increased physiological demands associated with child growth [14] and also, reduced intake complicated by predominantly cereal based diet with high content of phytates, phenols and other ligands that impair iron absorbtion [15]. Increased loss of blood from hookworm infestation has also been estimated to account for a significant proportion of iron deficiency anaemia [16] .

The high prevalence of anaemia in children under five in Ghana, particularly within the northern sector and the negative consequences on the cognitive and behavioural development is a severe public health threat, requiring urgent need for effective and efficient public health interventions to reduce the burden of anaemia. Reduction of the burden of anaemia requires multifaceted approaches which has been a challenge for many developing countries resulting in over-reliance on hospital based emergency management of anaemia where children often report too late to be saved. Cost implications for developing countries prevents adherence to universal iron supplementation recommended by WHO for areas with anaemia prevalence exceeding 40% [17]. It is therefore necessary to develop a cost-effective community based management approach that take in to consideration locally available and sustainable interventions to reduce the burden of anaemia in children under five. This study seeks to conduct a community based randomized control trial assessing the efficacy of a Locally available dietary (LAD) intervention that uses a Behavioural Change Communication (BCC) approach that emphasise the elimination of inhibitors of iron absorption from diet, promotion of enhancers of iron absorption and encouraging the intake of locally available iron and folate rich diets. This study would compare effects of LAD intervention in community based management of anaemia in children under five years.

**1.2 Statement of the Problem**

Anaemia continue to remain a public health problem affecting most developing countries and a threat to the lives of children under five years. Anaemia in children under five years is of critical concern because of its impairment of mental, physical, social and behavioural development in children under five which persist in later years even after correcting the anaemia. Unfortunately, anaemia is very prevalent in the northern region. The Ghana demographic health survey (GDHS) found the northern region to have the worst indicators for anaemia and stunting in Ghana with an anaemia prevalence of 82% in children under five years [18]. Anaemia have significant effect on the growth, health and social well-being of these children. The under five year period remains a critical period within which anaemia must be avoided to ensure optimal brain and physical development. Despite the numerous public health implications of anaemia in children under five, its prevalence remains a significant severe public health threat in most developing countries. In Ghana, almost 8 out of 10 children under five years are anaemic with 55% of children having at least moderate to severe anaemia [18]. Some regions in Ghana particularly the northern, upper east and upper west regions have almost nine out of every ten children anaemic [19]. Several studies, and WHO recommendations advocate universal iron supplementation, dietary diversification and home food fortification. However, these interventions alone have not significantly reduced the prevalence of anaemia particularly in developing countries. Cost implication precluding effective universal implementation could be blamed for the lack of success. However, high intake of inhibitors of iron absorption as well as low intake of food rich in enhancers of iron absorption coupled with low consumption of iron rich diet play a significant role in the persistent high prevalence of anaemia in children under five [20]. For example, polyphenols in tea could reduce the bioavailability of iron in diet to about 90% while certain enhancers such as ascorbic acid could increase the absorption of iron by several folds [21]. The diet of children in the northern region contains some of this food substances that inhibits absorption of iron. Also dietary practices results in the intake of food with low bioavailability of iron. This brings to light the need to incorporate behavioural change communication intervention that promotes the intake of locally available iron rich food and enhancers of iron absorption, as well as discouraging consumption of inhibitors of iron absorption. In our environment, no randomised control trial has evaluated the safety and efficacy of treating anaemia with or without behavioural change communication that emphasise the discouraging of inhibitors, and promotion of enhancers of iron rich diet on the prevalence of anaemia, physical and behavioural development of children under five years.

**1.3 Justification of the Study**

The prevalence of anaemia in children under five is alarming and there is the urgent need to reduce this burden. Iron supplementation programs have not achieved their full anticipated effects. Many children continue to die because of anaemia and several others live with countless developmental disabilities. Given the abnormally high prevalence of anaemia in children under five, and it’s associated enormous public health implications of developmental disabilities across the life span [22], better methods are needed to improve iron supplementation and urgently reduce the prevalence of anaemia within this age group. Also, recommended iron supplementation for the treatment of anaemia in children usually produce large increase in colonic iron because, typical iron absorption is less than 20% of dose ingested [23] . Non-absorbed iron could be harmful to children because of its role in modifying the gut microbiota with the resultant effect of increasing intestinal pathogens [24] . Increasing number of studies in children under five reports adverse effects with iron supplementation such as decrease growth, increased diarrhoea, interaction with other trace elements and increased inflammatory markers [25] . It is absolutely essential to explore new cost-effective ways of maximization iron absorption and improving safety of supplemented iron. The possible synergistic role of these LAD activities outlined with IFA supplementation has not been fully explored. A study that would fill this knowledge gap and provide highly rated evidence for successful implementation of LAD necessary for improved iron and folic acid supplementation must be a randomized control trial hence its use as the preferred methodology in this study.

**1.4 Hypothesis**

It is hypothesized that, children receiving LAD intervention plus iron and folic acid would have better improvement in haemoglobin status, serum ferritin and developmental outcomes compared to children taking only iron and folic acid.

Thus

**H_0_:** There would be no statistically significant difference in mean haemoglobin, serum ferritin and developmental outcomes between study arm 1 [Iron + Folic Acid only arm] and study arm 2 [Iron + Folic Acid + Locally Available Dietary intervention] after 12 weeks of interventions

**1.5 Research questions**

1. What is the baseline prevalence of anaemia and iron deficiency anaemia among children under five years in Kumbungu district?
2. What are the factors associated with anaemia among children under five years in the kumbungu district of Ghana?
3. What are the effects of LAD intervention in children aged 6-59 months receiving iron and folic acid for anaemia?

**1.6 Aim and objectives of the study**

**1.6.1 Aim**

To evaluate the safety and efficacy of Locally Available Dietary Intervention in combination with Iron Folic Acid in the community based management of anaemia in children under five years in the Kumbungu District of the Northern Region, Ghana

**1.6.2 Specific Objectives**

1. To determine the baseline prevalence of anaemia and iron deficiency anaemia among children under five years in the Kumbungu district of northern region.
2. To determine factors associated with anaemia among children under five years in the Kumbungu district of Ghana
3. To develop a Locally Available Dietary (LAD) intervention which is culturally acceptable, cost effective and nutritionally appropriate for a behavioural change communication
4. To test the effects of LAD intervention in children aged 6-59 months receiving iron and folic acid for anaemia.

**1.6 Conceptual Framework**

Several factors directly and indirectly affect the prevalence of anaemia in children under five. Political, socio-economic conditions, geography of the area where in children find themselves as well as socio-economic indicators of the country influences the prevalence of anaemia in a particular locality. Other factors include basic healthcare infrastructure and distribution of services. All these factors influence intermediate factors such as preventive and curative services, food diversity, environmental sanitation and hygiene, maternal and child health practices. The causes of anaemia such as iron deficiency and other micronutrient deficiency are results of the above.

**Figure 1.1: Conceptual framework; (source; developed based on findings from literature search)**

**CHAPTER TWO – LITERATURE REVIEW**

1. **Introduction**

Anaemia affects approximately a third of the world’s population [26] . It can be described as a state in which there is decreased levels of red blood cells or haemoglobin concentration in the blood of the individual consequently leading to insufficient supply of oxygen in the body [27, 28]. In the year 2013, a study on global, regional and national trends in haemoglobin concentration and prevalence for 1995 -2011 found out that, anaemia accounts for more than 68 million years lived with disability which is more than estimates combined for major depression, chronic respiratory diseases and injuries [6]. Anaemia is derived from the Greek words “an” meaning without and “haima" meaning blood [29].

- 1. **Classification of anaemias in children under five**

Anaemia has several classification systems. They are frequently classified by the cause but can also be classified by the cytometric features such as size, shape and colour of the red blood cells [30]. Some classification systems also incorporate erythrokinetic and biochemical parameters. Using the cytometric classification, anaemia can be classified as normocytic nomochromic, microcytic hypochromic and macrocytic normochromic as follows:

- Normochromic, normocytic anemia (normal MCHC, normal MCV).
  - anemias of chronic disease
  - hemolytic anemias
  - anemia of acute hemorrhage
  - aplastic anemias
- Hypochromic, microcytic anemia (low MCHC, low MCV).
  - iron deficiency anemia
  - thalassemias
  - anemia of chronic disease (rare cases)
- Normochromic, macrocytic anemia (normal MCHC, high MCV).
  - vitamin B_12_ deficiency
  - folate deficiency

Pathologically, anaemia can be classified as regenerative anaemia and hypo-regenerative anaemia [31] . In regenerative anaemia, reticulocyte count is high and it is characterised by increased production of erythropoietin in response to loss of red blood cells. Examples include haemolytic anaemia and anaemia from bleeding. Based on erythrokinetic viewpoint, anaemia could be classified as anaemia of haemorrhage, haemolytic anaemia and aplastic anaemia [29]. Also based on the clinical presentation, anaemia could be classified as acute or chronic

- 1. **Anaemia as a public health problem in children under five**

Anaemia is a widespread public health threat, affecting people of all ages, sex, and of all geographical regions. In the year 2010, the global prevalence of anaemia was estimated at 32.9% [14]. Among children aged five and below, the prevalence of anaemia globally was estimated at 43% in the year 2011 [6]. Severe anaemia is a major causes of sickness and death among children under five in sub-Saharan Africa [32].

- - 1. **Prevalence, Burden and distribution**

Global statistics for anaemia is not published in real time. The most recent publication of the global prevalence of anaemia dates back to a study conducted and published in 2013 which studied global haemoglobin concentration from the year 1995 to 2011 [6, 14]. In that study, the global burden of anaemia was estimated to affect more than 2.2 billion of the world’s population in 2010, thus 32.9%. The age group 0-5 years were most affected with a prevalence of 41.8%. In Africa, more than 60% of preschool children are anaemic [4]. The WHO African region had countries with the lowest haemoglobin levels and highest prevalence of anaemia. Using 2011 data, west Africa and central Africa together has a prevalence of anaemia in children under five years estimated at 71% with severe anaemia accounting for 5% [6]. The mean haemoglobin of children aged less than five years in the global, regional and national trends in haemoglobin study published in 2013 found children from central and West Africa to have the lowest mean haemoglobin concentration among children under five [6]. Significant inequalities exist in the anaemia burden between the wealthiest and the poorest quintiles in Ghana [33] .

- - 1. **Aetiologies of anaemia in children under five**

Majority of anaemia in children can be classified as nutritional anaemia. The most significant nutritional deficiency resulting in anaemia is iron. Almost half of all anaemia is due to iron deficiency anaemia [14]. Second most important cause of nutritional anaemias is vitamin B12 deficiency [34]. Iron is an important substance necessary for almost all living organisms. It forms part of a wide variety of metabolic processes, including oxygen transport, deoxyribonucleic acid (DNA) synthesis, and electron transport [21].

In humans, the body requires iron for the synthesis of its oxygen transport proteins, such as haemoglobin and myoglobin [35]. It is also required for the formation of heme enzymes and other iron-containing enzymes involved in electron transfer and oxidation-reductions [24] . The diets of infants and young children aged six months to 23 months generally provide insufficient amounts of key micronutrients (particularly iron, vitamin A, zinc and calcium) to meet their nutritional needs [36] . This coupled with increased demands of such nutrients due to growth and development of the children results in inadequate amounts to support the physiological demands for optimum growth of the child [37]. Also, the inclusion of animal-source foods to fill the nutrient gap may be impractical for low-income countries [36]. The WHO recommends exclusive breastfeeding until six months of age and continued breastfeeding for at least two years however certain cultural and social believes makes this difficult to achieve among many mothers in the developing world [38–40].

- - 1. **Determinants of anaemia in children under five**

Several factors have been attributed to anaemia. Broadly, there could be classified as biological, genetic, infectious and inflammation, environmental, social and behavioural determinants. Some other studies describe the following as factors associated with anaemia in children under five. Poor nutritional intake, low iron bioavailability, low folate and Vitamin B12 intake, lower age and poverty. Others include; less maternal education, increasing family size, less iron intake, bottle-feeding, prolonged breast-feeding without proper weaning, malaria infection, lack of maternal antenatal care, food insecurity, increasing birth order, less birth interval, low family/parental income and lack of adequate sanitation [41]

- - - 1. **Biological determinants**

Biological factors influence the development of anaemia in children under five years. Several studies have found association between the development of anaemia and several biological factors. Biological factors influences the development of anaemia either by decreasing the production of red blood cells or by increasing the loss or destruction of red blood cells [26]. The first group of factors that influences the development of anaemia include the nutrient related factors which leads to nutritional anaemias [41]. The most common causes of nutritional anaemia is iron deficiency which accounts for about half of all anaemias [26]. Other factors include; vitamins A, B6, B12, C, D and E. others include folate, riboflavin and copper. The lack of the above nutrients, either through insufficient intake or increased loss from parasites significantly contribute to anaemia. Also, excess intake of certain nutrients such as phytates also inhibit the bioavailability of this nutrients.

Other biological factors that contributes to the development of anaemia includes physiological state, growth, sex and age. Children below 2 years are at a greater risk of anaemia due to the combination of increased growth demands and sex. Studies in infants show consistently lower iron stores in males compared with female infants which suggest a potential hormonal effects in the development of anaemia [26].

- - - 1. **Genetic determinants**

Genetic factors such as sickle cell disease and thalassaemia are common causes of anaemia in children. Globally, 5% of all persons carry an inherited haemoglobin variant. This proportion is higher for Africa where 18% carry an inherited haemoglobin variant. Genetic determinants contribute to anaemia by haemolysis and suppressed haemopoiesis.

- - - 1. **Infectious and inflammation determinants**

Certain disease conditions have shown convincing effect on the prevalence of anaemia. Malaria and soil transmitted helminths are the common causes of anaemia of infectious origin among children [26]. The mechanism through which these infections act is by promoting nutrient loss, impairing nutrient absorption and or metabolism. The principal causes of anaemia from parasites include infection from soil transmitted helminths such as hookworm [26]. Heavy hookworm infection can lead to about 5mg per gram of faeces loss of iron. Another parasitic infection which is commonly implicated in blood loss in children and its associated anaemia is schistosomiasis. Schistosomiasis though eventually leads to blood loss, the exact mechanism could include spleen sequestration and hemolysis of red blood cells.

Malaria is the most common infectious cause in sub-Saharan Africa. In the year 2015, about 88% of global malaria case were from sub-Saharan Africa [42, 43]. About 15% of all anaemia in children under five are attributable to malaria [26]. Malaria from *Plasmodium falciparum* is the most cause of anaemia in children under five. The mechanism of causing anaemia is due to haemolysis of red blood cells and suppression of hemopoiesis through the process of upregulation of hepcidin which results in prevention of iron absorption from diet and the redistribution of iron to macrophages resulting in low availability of iron for hemopoisis [44]. Other disease conditions such as HIV infection and Tuberculosis contributes to anaemia.

- - - 1. **Socioeconomic and Environmental determinants**

Socioeconomic factors are strongly linked to anaemia in children under five. Poor socioeconomic status affects nutritional behaviours which contribute to anaemia. Poverty is a factor that affects health seeking behaviour and prevention of anaemia which increases the likelihood of a child becoming anaemic [41]. Also low maternal educational attainment is linked with child anaemia. Mothers with low/ no formal education are more likely to have children who are anaemic [45]. Educational status influences mother’s ability to process nutritional information and provide adequate nutrition for the child [46]. Rural residence is associated with higher prevalence of anaemia due to the often inequity of distribution of social amenities such that resident of urban areas are more likely to have access to services including health facilities, sanitation, water [26].

- 1. **Consequence of Anaemia**

Anaemia has serious consequence on health. It has been estimated that, anaemia accounts for about 9% of total disability burden from all diseases [47] Its consequence on health can be classified as medical, neurodevelopmental and economic consequence.

- - 1. ***Medical consequence***

Medical effect of anaemia is manifest in interference of physical growth and performance and influencing morbidity and mortality. The effect of anaemia on child mortality and morbidity is enormous. Child haemoglobin level 5.0g/dl and below is strongly associated with child mortality [26],. However the relationship with disease morbidity is complex partly because iron is essential for immune function.

Anaemia can negatively affect the physical performance and productivity of its victims as a result of reduced oxygen transport associated with anaemia and coupled with reduce cellular oxidative capacity associated with iron deficiency anaemia [14] .

- - 1. ***Child neuro-developmental consequence***

Although nutrition is important for the functioning of the brain throughout the lifespan, nutritional deficiency particularly in infancy affects brain development because it’s the period of most rapid brain development [48]. Prevention of anaemia in infancy promotes neuro-development [49]. Micronutrients such as iron are essential for key neurodevelopmental processes such as [48]: neuron proliferation, axon and dendrite growth, synapse formation, pruning, and function, myelination, and neuron apoptosis. The actual impact and the likelihood of permanent neurodevelopmental damages depends on several factors, including; the child's experience and input from the environment, the timing of nutrient deprivation, the degree of nutrient deficiency, and the possibility of recovery [48, 50]. While strategies such as micronutrient powders have been shown to improve these conditions, direct evidence of their impact on brain development is scarce and when available inconclusive [48]. This calls for promotion of behavioural feeding habits early in life so as to prevent this micronutrient deficiency and its attendant neurodevelopmental effects. Also, persistent neurocognitive changes in spite of iron repletion have augmented the significance of avoidance and early detection of iron deficiency [51].

- - 1. ***Economic consequence***

The long-term consequences of anaemia are not only seen at the individual level but also have deleterious impacts on the economic development and human capital at the country level [4]. A significant proportion of the world’s poor are caught in nutrition-based poverty traps that make them likely to remain poor over generations [52]. This effect is largely due to decrease in oxygen in blood effect of anaemia that reduces the aerobic capacity of anaemics with a resultant effect of reduced physical, mental function [52]. This affects productivity at school which in turn affects future earnings maintain the cycle of poverty.

- 1. **Clinical presentation**

Anaemia in children under five could present with varied signs and symptomatology as shown in table 2.1. Very frequent signs and symptoms include paleness which is a finding in almost half of all anaemic children. Other symptoms include dyspnoea, easy fatigability, tachycardia, cardiac murmurs.

Table 2.1; Signs and symptoms of anaemia

| **Very Frequent Signs and symptoms** | **Rare signs and symptoms** |
| --- | --- |
| Paleness (45–50%)  Fatigue (44%)  Dyspnoea  Headache (63%) | Haemodynamic instability (2%)  Syncope (0·3%)  Koilonychia  Plummer-Vinson syndrome (<0·1%) |
| **Frequent Signs and symptoms** | |
| Diffuse and moderate alopecia (30%)  Atrophic glossitis (27%)  Restless legs syndrome (24%)  Dry and rough skin  Dry and damaged hair  Cardiac murmur (10%)  Tachycardia (9%)  Neurocognitive dysfunction  Angina pectoris  Vertigo | |

Source: adapted from Lopez et al (2016), Iron deficiency anaemia, The Lancet, 387, 10021

- 1. **Risk factors**

Physiological demands for iron in children predispose children to anaemia.

A recent study based demographic health survey data, found that demographic factors, environmental factors, socioeconomic factors, family structure, water/sanitation, nutrition and growth, maternal factors, recent illnesses contributed to anaemia among young children in sub Saharan Africa [53].

- 1. **Diagnosis of anaemia**

The WHO currently defines anaemia as having a haemoglobin concentration below 110 g/L or 11g/dl in children 6–59 months. These Haemoglobin cut-offs were estimated based on the fifth percentile of the haemoglobin concentration of a normal population [17]. Measurement of serum ferritin, transferrin saturation, serum soluble transferrin receptors, and the serum soluble transferrin receptors–ferritin index are more accurate than classic red cell indices in the diagnosis of iron deficiency anaemia [14, 54]. Due to the cost of serum iron studies, most developing countries are not able to apply it universally for anaemic children. This resulted in the assumption that all anaemia is as a result of iron deficiency requiring the supplementation of iron. Recent studies have shown the usefulness of reticulocyte haemoglobin content (CHr), serum transferrin receptor (sTfR) and sTfR/log ferritin (sTfR-F index) parameters to diagnose iron deficiency without anaemia using specific cut-off points [54, 55] .

- 1. **Inhibitors of iron absorption and enhancers of iron absorption**

Certain factors improve iron absorption. Ascorbate and citrate increase iron uptake in part by acting as weak chelators to help to solubilize the metal in the duodenum [56, 57]. Iron is readily transferred from these compounds into the mucosal lining cells. The enhancing effect is largely due to its ability to reduce ferric to ferrous iron but is also due to its potential to chelate iron. Ascorbic acid will overcome the negative effect on iron absorption of all inhibitors, which include phytate, polyphenols, and the calcium and proteins in milk products, and will increase the absorption of both native and fortification iron [57]. The enhancing effect of meat, fish, or poultry on iron absorption from vegetarian meals has been shown, and 30 g muscle tissue is considered equivalent to 25 mg ascorbic acid [58]. Addition of chicken, beef, or fish to a maize meal increased non-heme iron absorption 2-3-fold.

In plant-based diets, phytate (myo-inositol hexakisphosphate) is the main inhibitor of iron absorption [59]. The negative effect of phytate on iron absorption has been shown to be dose dependent and starts at very low concentrations of 2-10 mg/meal. Polyphenols occur in various amounts in plant foods and beverages, such as vegetables, fruit, some cereals and legumes, tea, coffee, and wine. The inhibiting effect of polyphenols on iron absorption has been shown with black tea and to a lesser extent with herbal teas [21]. Tea can reduce iron absorption by 90% [14].

The WHO advocates food based approaches at managing anaemia [26]. The WHO promotes the consumption of iron rich foods such as meat, fish, poultry, organs from cattle and fowl. Plant based foods recommended by WHO includes legumes and green leafy vegetables [14].

- 1. **Interventional strategies to address anaemia in children under five**

Many studies agree that, Nutritional anaemia should ideally be addressed through dietary diversification and improved access to foods that have high iron bioavailability, including animal products [6, 60]. However, anaemia in children under five can be addressed with the appropriate supplement or food fortification, these programs struggle to reach the poorest, out-of-school children [48]. Firstly, micronutrient supplementation are the treatment of choice in managing anaemia particularly in the clinic setting [61]. Food fortification is a target approach used in some settings. It is effective provided the fortified foods is easily accessible, affordable and available at the local distribution network [62]. Dietary diversification and enhancement of micronutrient absorption, deworming, antimalarial and water and sanitation interventions that help prevents diarrhoea in children have been found to be effective at reducing the burden of anaemia in children under five years.

- 1. **Deworming as an adjunct to iron and folic acid in the management of anaemia**

Several studies has shown the effect of anthelminthic in improving the haemoglobin of children [63, 64]. It is therefore believed that the presence of infectious causes of anaemia would often limit the efficacy of supplemented iron [65]. The WHO recommends preventive chemotherapy (deworming), single-dose albendazole (400 mg) or mebendazole (500 mg) as a public health intervention for all young children 12–23 months of age, preschool children 1–5 years of age using annual or biannual schedule if the baseline prevalence of any soil-transmitted infection is 20% to 49% or over 50% respectively [66–68]. The aim is to reduce the burden of soil transmitted helminth infection and prevent the myriads related complications with soil transmitted helminths including anaemia in children under five [66]. A half-dose of albendazole (i.e. 200 mg) is recommended for children younger than 24 months of age [69].

- 1. **Outstanding research questions**

Studies on anaemia in children abound, however, studies within the specific area of locally available dietary intervention still remains lacking. Most of the recommendations from expert groups on the use of locally available dietary interventions have not been subjected to rigorous randomised control trial. It is the hope that this knowledge gap would be filled as this study evaluates the efficacy of locally available dietary interventions in improving anaemia in children under five years.

**CHAPTER THREE – METHODOLOGY**

**3.1 Study Site**

The study would be conducted in the Kumbungu district of the Northern region. Kumbungu means “I have killed my donkey”. The northern region was selected because, it has one of the highest prevalence of anaemia in children under five. Several activities by Non-Governmental Organizations (NGOs) at addressing the problem of anaemia have shown little improvement. The Northern region has 26 districts. The Kumbungu district has been chosen for the study because of its high prevalence of anaemia in children under five.

Kumbungu district is one of the smallest districts within the northern region with a total landmass of 1599 Square Kilometres. Kumbungu has 39,341 inhabitants within 4,133 households and a sex ratio of 100.2 males per 100 females. Children constitute 48.4% of the population. 7,101 of the population of Kumbungu are aged 0 to 4 years [70]. Average household size in Kumbungu is 9.5. The total fertility rate, crude birth rate and general fertility rate for the district are respectively 3.6, 23.4 per 1000 and 103.9 births per 1000 women aged 15 – 49. The household structure is mainly that of extended family system accounting for 71.8% of the household population while nuclear family system accounts for 14.6%. According to the recent census, only 15.2% of the population is literate in English language and 63.8% of persons aged 3 years and older in the district have never attended school. With regards to employment status, only 1.7% of the total population are employees. Majority of the working population are in farming related occupations. The district has 115 communities, most with a population below 500. Only two communities have population above 5000. With regards to housing, 79.4% of the inhabitants in the district live in thatch/palm/raffia roofed houses [70]. The district houses the only major water treatment in the northern region.


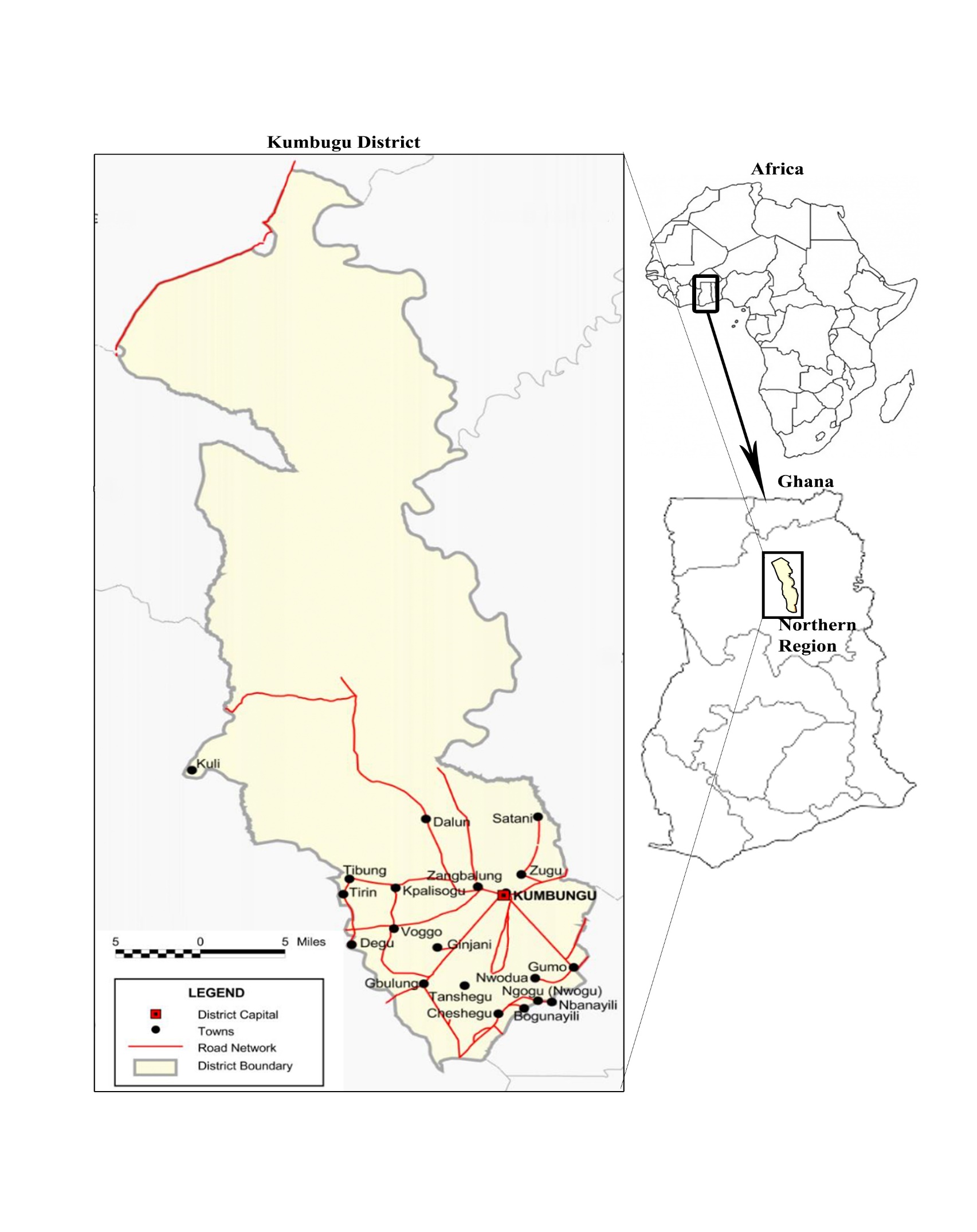


Figure 3.1: Map showing Kumbungu district

**3.2 Study design**

This study will be a community-based cluster randomized, controlled trial, with two parallel arms. Included communities shall be randomly selected. Cluster assignment is at the level of household for the locally available dietary (LAD) intervention. The study would follow the closed cohort of eligible households with children aged between 6 to 56 months at baseline for 12 weeks. All eligible children within the household would be included in the study. The interventions would be administered throughout the 12 consecutive weeks of the study. Primary outcome data would be collected at baseline (pre-intervention) and then at the end of 12 weeks (post intervention). Eligible children within the household are the unit for analysis and the clustering would be taken into account.

Cluster randomization was chosen for practical reasons and also to minimize contamination. Children of the same household eat from the same pot. Since the study assess behavioural change communication targeting intake and avoidance of enhancers and inhibitors of iron absorption, it was more appropriate to randomized at the level of the cluster, instead of individual randomization. LAD intervention would be administered at household cluster level. Households must have at least a child aged 6 – 56 months at baseline.

***3.2.1 Study Arms***

1. Iron + Folic Acid (IFA arm): Standard care arm
2. Iron + Folic Acid + Locally available dietary intervention (IFA+LAD arm): Standard Plus

**3.3 Interventions**

Intervention pertains to both cluster and individual level. At the cluster level, randomised household would each received targeted LAD intervention aimed at promoting the intake of locally available iron, folate and vitamin B12 rich foods, food rich in enhancers of iron absorption and discouraging the intake of food rich in inhibitors of iron absorption. The rest of the interventions are targeted at the individual participant level. All study participants would receive the current practiced standard treatment for anaemia; iron + folic acid.

The standard care arm would receive only the iron plus folic acid syrup. The standard plus arm (arm two) would receive iron, folic acid syrup treatment plus LAD intervention. All two arms of the study would run parallel and would be randomized in the ratio 1:1. The dosage of Iron and folic acid to be dispensed would be according to the current WHO recommended dose for age of the child [26].

Each study participant is required to take the prescribed doses daily. Monthly iron and folic acid requirement shall be dispensed to mothers/guardians. A member of the study team shall visit each household every week to monitor the administration of the iron and folic acid. Behavioural change communication administered at household level shall include a set of five points counselling guideline promoting the intake of food rich in enhancers of iron absorption and discouraging the intake of food poor in enhancers iron absorption. This guideline would be administered monthly to randomized households in a LAD arm. In all mothers/guardians of households randomized for LAD would receive three monthly BCC counselling. A monthly semi-quantitative assessment of intake of iron, folic acid and vitamin B12 intake as well as known inhibitors/enhancers of iron absorption of iron intake shall be determined at baseline (week 0), and then weeks 4, 8 and 12.

**3.4 Description of Study Population**

Study participants are children aged 6 to 56 months. Children less than six months are expected to be exclusively breastfed according prevailing guidelines. Promoting behavioural change communication that promotes intake of enhancers of iron absorption and avoiding the intake of inhibitors of iron absorption would not be appropriate in the below 6-month age group. Participants must also be less than 56 month of age at baseline because the intervention is expected to last for three months and it is expected that, participant recruited must be below five years at post intervention. Children within selected household 6 to 56 month at baseline are eligible for inclusion in the study.

Study participants above three years may be enrolled in a preschool. Enrolment in a pre-school does not affect inclusion in the study. However, data on pre-school enrolment would be collected. Most of the study participants are from poor resource setting. Both males and females have equal chance of inclusion in the study. Typically, each household consist of about 9 family members of which children below five years constitute about 2 per household.

**3.5 Inclusion and Exclusion criteria**

***3.5.1 Inclusion criteria***

***3.5.1.1 Inclusion criteria at cluster level***

At the cluster level, household inclusion to the study must satisfy the following:

1. Household within the selected community.
2. The household must have at least a child aged 6 to 56 months.

***3.5.1.2 Inclusion criteria at the participant level;***

1. All children within the eligible household aged 6 to 56 months at baseline.
2. Haemoglobin concentration measured at baseline should be less than 11.0 g/dl.
3. The child should be residing in the selected community for at least the past three month.
4. Must have a legal guardian capable of providing informed consent.

***3.5.2 Exclusion criteria***

Eligible children with any of the following would be excluded from the study:

1. Current infective illness (example; respiratory infection, diarrhoea) with fever; however, children may be rescreened again after recovery if otherwise eligible. Current infective illness would be assessed from self-reported and verification of available medical records.
2. Diagnosed case of any clinical haemoglobinopathy (eg, beta-thalassemia major, HbE-beta thalassemia, Sickle cell disease). This will be assessed based on self-report and available medical and laboratory records.
3. Received iron supplements or iron-containing MMP in the previous month.

**3.6 Definition of variables of the study**

For the purpose of this study, anaemia would be defined according to the World Health Organization’s (WHO) haemoglobin level to diagnose anaemia in children 6 to 59 months at sea level. Based on the classification; haemoglobin level less than 110 grams per litre (11 grams per decilitre) is considered as anaemia. Mild, moderate and severe anaemia are respectively defined as haemoglobin level range of 10.0 to 10.9, 7.0 to 9.9 and lover than 7.0 grams per decilitre (g/dl) [17]. A household is as defined by the Ghana statistical service; a person or a group of persons, who live together in the same house or compound and share the same housekeeping arrangements.

**3.7 Sample size calculation**

The sample size was calculated to determine the minimum difference in prevalence of anaemia between arms of the study. The following information was used:

1. Estimated prevalence of anaemia at baseline is 82%, and an estimated 20% reduction after 12 weeks.
2. Estimated average cluster size; which is the average number of children per household is 1.7 (≈2) obtained by the ratio of number of children aged less than five years (7,101) to the number of households in the district (4,133).
3. An estimated intra-cluster correlation (ICC) of 0.010
4. Estimate of coefficient of variation of cluster sizes of 0.90
5. Power of at least 80% (0.80)
6. Significance level of 5% (0.05)
7. Allowing for a cluster randomization design effect of 1.03 and
8. A dropout rate of about 5% for the 12 weeks period,
9. A contamination effect of 10%

With the above considerations, and using a menu driven facility for sample size calculation in cluster randomized controlled trial [71] available as add-on in STATA (version 11.1, StataCorp, Special Edition, College Station, Texas 77845 USA), the minimum sample size and number of cluster per arm was calculated. The computation made use of the following formula for calculating the number of clusters (households) [71]:

$$k=1+\frac{n_{i}}{m}+\mathrm{CVIF}$$

Where

$$\mathrm{CVIF}=\frac{{cv}_{clusters}^{2}\left( \mu_{1}^{2}+ \mu_{2}^{2} \right)\left( z_{\alpha/2}+z_{\beta} \right)^{2}}{d^{2}}$$

And

k = number of clusters required per study arm

$n_{i}$= number of sample size required under individual randomization

*m* = number of clusters required per arm

*d* = pre-specified difference

${cv}_{clusters}$= intra-cluster correlation (ICC) coefficient

$z_{\beta}$ =Maximum available power

$z_{\alpha/2}=$Denotes the upper ${100}_{\alpha/2}$ standard normal centile

$\mu_{1} and \mu_{2}$ = mean/proportion per study arm

The minimum sample size per arm is 92 and the number of clusters per study arm is 54 households (minimum sample size/ average cluster size= 92/1.7). Considering this study proposes two parallel arms, the total minimum study sample size is 184 and the minimum number of households is 108 households.

**3.8 Sampling Process**

A two-stage multi stage sampling approach will be used as follows:

***3.8.1 First stage: Community selection***

- All communities in the district would be eligible for selection
- A simple random sampling technique of balloting without replacement would be used to select four communities
- In the event that the selected communities are not large enough to give the desired number of at least 108 households and 184 children under five, a simple random technique would be employed to select additional communities until the desired number of household and children are achieved.
- In the event that the communities have more than the desired number of households with eligible children when put together, a quota system would be used to allocate the number of household to be selected per each of the four selected communities.
- In case of large communities, the community would be stratified and uniformly sample from each stratum.

***3.8.2 Second Stage: Household and individual selection***

The process of obtaining households will be sampled based on the following guidelines:

1. Conduct a quick House and Household Listing in all four selected communities
2. Survey eligible households with the selected village
3. Interview only Household Heads/Spouse (Care givers) per each household
4. All eligible children per selected household would be enumerated and included in the study

At the end of the process a total sample size of at least 184 children and 108 households will be sampled from at least, four communities across the targeted district.

**3.9 Recruitment of study participants**

Kumbungu district has a 115 communities most of which are farming communities with population below 500 inhabitants and an average of 62 under-five per community. About six communities would be selected by simple random techniques and the a census of under-five within all households obtained. Community entry procedures would be followed and community leaders, assemblyman, and/or chief involved in a stakeholder engagement process. A selected household head and members of the household would be educated on the study procedures and consent obtained from the head of the family. Accent would be obtained from the individual children taking part in the study.

**3.10 Randomization: sequence generation, stratification and blocking**

The data from the baseline survey would be used for sequence generation. Clusters would be stratified by mean household haemoglobin. Using the mean household haemoglobin, the clusters would be stratified into Level I; 10.0 g/dL ≥ Hb ≤ 10.9 g/dL, Level II; 7.0 g/dL ≥ Hb ≤ 9.9 g/dL, and Level III Hb < 7.0 g/dl

Households would be ordered alphabetically using household head’s surname. The ordered alphabetical name would be imported in to a statistical computer software STATA V.11 at the community health department, school of public health, University of Ghana. The program would generate a series of blocks of varying size (four, eight and twelve) for each stratum and allocate the household clusters to study arm randomly within each block [72]. Consent to randomize would be obtained at the cluster level from the household head.

**3.11 Allocation concealment mechanism and blinding**

The treatment allocation for each household would be kept in the community health department until the study sites has completed training and are ready to implement the intervention. Clusters would be identified and recruited before randomisation. All eligible patients within the cluster would be included in the study. Allocation would be concealed from the community health nurses and family head who gave consent for household to be included in the trial. Also, allocation sequence would be concealed from researchers/clinicians enrolling and assessing participants. Sequence would be concealed in a sequentially numbered, opaque sealed and stapled envelopes. Corresponding envelopes would be opened only at the time of allocation of intervention. The intervention would be prepacked and correspond to the study arm assigned to. Independent clinicians that would assess outcome would be kept blind to intervention assignment of the household.

**3.12 Outcome measures**

Outcome measures mainly pertain to individual participant level. The study will evaluate the effect of LAD interventions on the prevalence of anaemia in the study arm.

***3.12.1 Primary outcome***

The primary outcome measures are mean haemoglobin levels and prevalence of anaemia in study arms. The haemoglobin level would be measured using a haematological analyser that measures the full (complete) blood count. Haemoglobin would be measured at baseline and end of intervention. Mean haemoglobin concentration (Hb g/dL) and prevalence of anaemia in children: Hb <11.0 g/dL; mild anaemia (10.0 g/dL ≥ Hb ≤ 10.9 g/dL), moderate anaemia (7.0 g/dL ≥ Hb ≤ 9.9 g/dL), and severe anaemia as Hb < 7.0 g/dl would be determined at baseline and at end of intervention.

**3.12.2 Secondary outcome**

A number of secondary outcomes would be measured in this study. Secondary outcomes would include measurement of anthropometry, and dietary recall, serum iron, ferritin, and other parameters of full blood count.

***3.12.2.1 Anthropometric Measures: Physical growth***

Secondary outcomes pertaining to child physical development would be measured and compared among study arms. The change in prevalence of underweight (weight-for-age, Z-score <−2), wasting (weight-for-height, Z-score <−2), stunting (length/height-for-age, Z-score <−2) and Mid Upper Arm circumference (MUAC) for age would be measured. Others measures include head circumference for age, and body fat composition estimates using triceps skin fold thickness (TSF) for age and subscapular skin fold (SSF) for age. Body weight would be measured to the nearest gram using a battery powered digital scale; it would be measured under standardized conditions (patients dressed in underclothes, barefoot, and with an empty bladder). Height or length would be measured using stadiometer or infantometer to the nearest millimetre. Height would be obtained for children above 2 years and length for children below 2 years. Head circumference would be measured using a flexible non-elastic tape measure. The tape measure would be wrapped snugly around the widest possible diameter of the forehead; about 1-2 fingers above the eyebrow to the widest part of the back of the head.

The MUAC is the circumference of the left upper arm. The following steps would be employed to measure it: Bend the left arm, and find the mid-point between the olecranon process and acromion. With the arm hanging straight down, a MUAC tape would be wrapped around the arm at the midpoint mark. MUAC would be measured to the nearest 1 mm. Triceps and subscapular skinfold thickness would be measured from the left side of the body to the nearest 1 mm using a skinfold calliper at the following sites: triceps skin fold, halfway between the acromion process and the olecranon process. The subscapular skinfold thickness, would be measured approximately 20mm below the tip of the subscapular skinfold. All anthropometric measurement would be measured twice and the average taken. The WHO growth standards Z scores provide a technically robust set of tools to describe the growth of children under five years of age. Its development involved studies in six countries of which Ghana was among. This makes it ideal for use in Ghanaian population hence it would be the reference chart used for all anthropometric measurement.

***3.12.2.2 Biochemical outcome measures***

The main biochemical measure would be the serum ferritin for all the study participants at baseline and at the end of the intervention period. Serum ferritin levels would be measured using a chemistry analyser employing immunoturbidimetric assay method and would be reported in micrograms per litre. Serum iron concentration and serum transferrin saturation would also be determined. Serum Vitamin B12 and serum folate levels will also be determined at baseline and at end of the intervention period.

***3.12.2.3 Clinical assessment***

Clinical assessment would be carried out at baseline and at the end of the study. It would include a general examination; oedema, pallor, jaundice, skin colour and hair changes. Systemic examination conducting cardiovascular, respiratory and abdominal examinations would be carried out looking out for abnormalities on examination. Other specific examination for micronutrient deficiency would be carried out. For vitamin A deficiency; night blindness, Xerophthalmia, conjunctival xerosis, corneal ulceration, keratomalica. A clinical assessment checklist would be used to capture data regarding clinical assessment. A clinical diagnosis of nutritional status would be made for each study participant.

**3.12.2.4 Dietary recall and quantification of dietary content of micronutrients**

A monthly 24-hour dietary recall would be measured for all study participants. It would be structured to provide requisite information for quantifying the World Health Organization indicators for assessing the quality of infant and young child feeding which include: timely introduction of complementary foods; frequency of feeding; diversity; both frequency and diversity); intake of vitamin A–rich foods; and intake of iron-rich foods. The above is expanded to include the locally available food rich in enhancers and inhibitors of iron absorption. It will also estimate the content of vitamin B12, and folate in the diet using the aid of a food composition tables and detail 24 hour recall [73]. The 24-hour recall would also have a section that estimate the recipes in the locally available food taken so as to obtain a fairly representative content of micro nutrients in the meals eaten. It will be structured to also determine nutrient adequacy ratios of meals in the 24hour dietary recall.

**3.12.2.5 Other haematological measures**

Other haematological indices such as mean corpuscular haemoglobin (MCH), mean corpuscular volume (MCV), Mean corpuscular haemoglobin concentration (MCHC) will be measured. The white blood cell count and its differentials would be obtained in a complete blood count test.

***3.12.2.8 Household food insecurity access scale (HFIAS) for measurement of food Access***

Household food insecurity may be a confounder that may influence adoption of behavioural change communication intervention promoting intake of enhancers of iron absorption and discouraging the intake of inhibitors of iron absorption. A nine-item food insecurity scale, HFIAS developed by the USAID’s FANTA project [74], as a universal measure of food insecurity would be used. The instrument has measures that begins with anxiety about food supply, decrease in quality of food, decrease in quantity of food, going to sleep hungry, and going all day and all night without food. It is scored on a scale of 0 to 27 where a higher number means a household experience more food insecurity. The assessment would be carried out at baseline and the end of the intervention.

**3.12.2.9 Household dietary diversity score**

Household dietary diversity score (HDDS) is a Food and Nutrition Technical Assistance (FANTA) III project that measures the number of food groups consumed over a given reference period [75, 76]. Dietary diversity is a qualitative measure of food consumption that reflects household access to a variety of foods, and is also a proxy for nutrient adequacy of the diet of individuals. It is an important indicator that describe the diversity of diet consumed by a household or individual in the past 24hours. This study would use the dietary diversity tool in relation to individual level. Dietary diversity scores have been validated for several groups as a proxy measure for macro and micronutrient adequacy of the diet. A more diversified diet has been associated with a number of improved outcomes in child anthropometric status, improved haemoglobin concentration and developmental scores. The data is collected by asking the respondent a series of yes or no questions addressed to the person in charge of food preparation. ‘No’ is scored ‘zero’ and ‘yes’ scored ‘one’. The sum of the HDDS ranges from 0 to 12. The mean HDDS would be computed per study arm at baseline and at the end of the intervention. Appendix 4 shows the HDDS study tool.

**Table 3.1 Baseline and Outcome measures, intermediary factors and time points**

| **Primary and Secondary outcomes** | | **Baseline** | **Week** | | **End-line** |
| --- | --- | --- | --- | --- | --- |
|  |  | **Week 0** | **4** | **8** | **Week 12** |
| **Primary outcome** | |  |  |  |  |
| 1 | Haemoglobin concentration | X |  |  | X |
|  | Anaemia prevalence | X |  |  | X |
| **Secondary Outcomes** | |  |  |  |  |
| **2** | **Anthropometry** |  |  |  |  |
|  | Weight | X |  |  | X |
|  | Length/Height | X |  |  | X |
|  | Mid Upper Arm Circumference | X |  |  | X |
| **3** | **Biochemical outcome** |  |  |  |  |
|  | Serum Ferritin | X |  |  | X |
|  | Serum Iron concentration | X |  |  | X |
|  | Serum transferrin saturation | X |  |  | X |
|  | Serum Vitamin B12 level | X |  |  | X |
|  | Serum folate | X |  |  | X |
|  |  |  |  |  |  |
| **4** | **Clinical Outcome** |  |  |  |  |
|  | ***General Examination*** |  |  |  |  |
|  | Oedema | X |  |  | X |
|  | Jaundice | X |  |  | X |
|  | Pallor | X |  |  | X |
|  | Skin and hair changes | X |  |  | X |
|  |  |  |  |  |  |
| **5** | **Dietary recall and quantification** |  |  |  |  |
|  | 24 hour dietary recall | X |  |  | X |
|  | Enhancers of iron in diet | X | X | X | X |
|  | Inhibitors of iron in diet | X | X | X | X |
| **6** | **Household food insecurity access scale (HFIAS)** | | |  |  |
|  | Household food insecurity | X |  |  | X |
| **7** | **Household dietary diversity score (HDDS)** |  |  |  |  |
|  | Administered at individual level | X | X | X | X |
| **8** | **Household Questionnaire** |  |  |  |  |
|  | Demographic data | X |  |  |  |
|  | Socioeconomic status | X |  |  |  |
| **9** | **Adherence to Study medication** |  | **X** | **X** | **X** |

**3.13 Procedure for Data collection**

A team of trained data collectors would carry out data collection at baseline and end-line. The data collection team would be made up of a physician specialist, optometrist, medical laboratory scientists, nurses and trained nutritionist. The behavioural change intervention would be administered by trained community health nurses who would each be responsible for each community. A three day training would be carried out before baseline assessment and a one-day refresher training prior to end-line assessment. Baseline data and end-line data collection is each estimated to take a week at week zero and thirteen respectively. The data collection team would visit households and administer household questionnaires as well as individual child questionnaires after completing consent and assent procedures. Clinical examinations would be carried out by the physician specialist, the optometrist carry and ophthalmic nurses carry out the eye examination.

A trained phlebotomist would carry out the bloodletting. The nutrition team would carry out the anthropometric assessment, dietary recall and quantification of dietary content of iron, folate, enhancers and inhibitors of iron absorption. Five millilitres of blood samples would be taken for haematological and biochemical analysis. The trained community health nurses would carry out the behavioural change communication intervention at week one, four and eight of the intervention period. Administration of pre-packaged and labelled iron and folic acid syrups would be administered by the community health nurses.

The community health nurses would not be involved in the data collection. The independent data collection team would be blinded to the household randomisation. Other information to be collected would include household characteristics, medication history, details about intake of IFA supplements, adverse events of intervention, out of pocket expenses for adhering to interventions, morbidity and mortality outcomes.

**3.14 Project Management**

***3.14.1 Reconnaissance visit***

A team led by the PI will conduct a reconnaissance visit to the six targeted communities. This visit will afford the team a good understanding of the project communities. This will also give the PI the opportunity to improve the proposed sampling and field strategies, as well as establish critical contacts with key stakeholders in the project communities.

***3.14.2 Development of Field Management Plan, Training Plans and Manuals***

Based on the outcomes of information obtained from the reconnaissance survey, the study will develop Data Collection Management Plan – which will include deployment of staff, data collection process and supervision plans. This plan will further be broken down into instructional materials for both supervisors and interviewers on the project. In addition, a Training Plan and Manual to guide the training of enumerators would be prepared. Questionnaires will be programmed onto a computer-based platform to allow for data collection using tablets or phones. The tablets will be used in the training of enumerators and pre-testing of quantitative tools.

***3.14.3 Recruitment of Interviewers***

About Ten enumerators/ field staff would be selected for the training (Eight will be selected for the study). The basic criterion for the recruitment of these individuals are a minimum of a Higher Diploma or University Degree in Nursing, Nutrition, and Medical Laboratory sciences or related fields. Other criteria would include experience in similar assignments, experienced in conduct of participatory research, and mastery of widely spoken local languages within the targeted district.

***3.14.4 Training of Field Staff and Pre-testing***

Based on the Training Plan, a centralized training will be organised to ensure standardisation of delivery and understanding by field staff. The training programme will be scheduled over a period of 5 days (inclusive of a 1 day for pre-testing). A joint session will be organised for all participants on the first day of training, after which they would be trained in their respective teams. This session will focus on general issues as follows:

- General issues including the project background, data collection plan and deliverables, research ethics, quality assurance procedures among others.
- Understanding of the questionnaires and guides
- Administration of Quantitative Questionnaires
- Attitudes and behaviours for field data collection among others

Training on the second and third days will be administered in three groups, namely,

1. Quantitative Class, which will consist of quantitative enumerators
2. Laboratory and phlebotomy class
3. Nursing/ follow-up class

In the group sessions, the training will focus on the data collection and study protocols

***3.14.5 Pre-testing of Tools and Skills***

Following three (3) days of classroom training, the field teams will be sent to one of the communities in the District, however not sampled to test their understanding and skills for the survey, and adequacy of the tools. On the fifth day, the teams will reconvene at the training centre to discuss the findings and field issues.

***3.14.6 Mobilisation of Logistics and Equipment***

The study will also use the time to mobilise logistics in preparation for the training and implementation of the field work. As a minimum, the following provisions will be made for the study; a 4x4 cross country vehicle, laptops, Mobile Tablets (for quantitative teams), external hard drive and pen drive, printed copies of questionnaire and guidelines, notepads and pens, name tags and introductory letters among others.

***3.14.7 Finalisation of tools and Upload on Survey Platform,***

The synthesized feedback will be used to update the tools for study. Once data feedbacks are used to update the quantitative tools (Household and Structure Observation Checklist) questionnaires will be uploaded to the survey platform and then downloaded to the mobile tablets.

**3.15 Description of study tools**

An electronic data collection system would be used in this study. Anthropometric measurement would be carried out using an electronic scale, stadiometer or infantometer depending on the age of the study participant, a shakier tape would be used to measure Mid Upper-Arm Circumference. A non-elastic tape measure would be used for other anthropometric measurements such as head circumference and abdominal circumference.

***3.15.1 Electronic data collection tool***

Data would be captured using an android seven-inch tablets with application programmed to suit the data to be collected. The programme would help in navigating the data collection team through a step-by-step process while interviewing household heads, mothers and making assessment and other measurements on study participants. All required information to be collected at each visit would be highlighted and the data collected guided through each step using a user-friendly interface.

The android application would have some data validation rules to prevent errors during data collection. Some of the rules would include logical and checks on acceptable ranges for each variable. It would also include skipping rules and guidelines and reminders on data that must be collected and entered. All data collection team members would have to log in using a pre-assigned unique server ID and password. Data collectors would be required to enter all required registration information of the child into the system and would be able to access information to which the respective data collector is assigned.

During the subsequent visits, the application would provide each data collector the list of children and households on which follow-up is required. The application would use a geographical information system data collected at the point of follow-up to verify physical presence at the data collection point. Stored data on the local android device would be uploaded to a central server. A backup copy of the database would be stored on a different server and daily updated.

***3.15.2 Questionnaire***

The questionnaires would be available on the android application and the data collector guided to complete the questionnaires. A demographic and household questionnaire would be administered at baseline of the study. Other questionnaires include a household questionnaire, and individual questionnaire for all children participating which would be completed by the primary care-giver or mother.

**3.16 Development of Locally available Dietary intervention for behavioural change communication**

The Locally available dietary intervention to be developed would be adapted from the WHO guide for dietary diversification and enhancing bioavailability of absorbed micronutrients. The guide has five points which would be locally adapted and include locally available food examples. The locally available foods would be selected with expect consultation and use of food composition table.

**Table 3.2** **WHO guide for food diversification and enhancing bioavailability of absorbed micronutrients**

| **WHO guide for food diversification and enhancing bioavailability of absorbed micronutrients** |
| --- |
| - Increase the production and consumption of iron-rich foods, primarily animal-source foods such as meat (especially red meat), poultry and fish, but also iron-rich plant sources such as legumes. - Increase the production and consumption of foods that are rich in vitamin A/carotenoid, such as green leafy vegetables, orange-fleshed fruits and vegetables (e.g. orange-fleshed sweet potatoes), dairy products, eggs, liver and fish oils. - Add fruits and vegetables that are rich in citric or ascorbic acid (e.g. citrus fruits) to the diet, to increase the absorption of non-haem iron. Vitamin C degrades with cooking, so consumption of uncooked (or lightly cooked) fruits and vegetables with high vitamin C content should be encouraged (assuming considerations of food hygiene and food safety are addressed). - Identify and promote culturally appropriate and feasible methods of food processing and preparation, to improve bioavailability and absorption.   *Iron*: germination, fermentation and soaking may improve absorption.  *Vitamin A*: short cooking times and steaming rather than boiling will maintain pro-vitamin A activity.   - Avoid combining known inhibitors of iron absorption with meals that are high in iron content; for example: separate tea and coffee drinking from meal times; consumption 1–2 hours later will not inhibit iron absorption; consume dairy products (milk, cheese and other foods made from milk) as a between meal snack, not at a meal time |

**3.17 Ethical consideration**

Written informed consent would be sought at the cluster level from the head of the household. Verbal assent would be obtained from individual study participant. The consent would be obtained at the baseline before randomisation into the study arms. The proposal would be submitted to the Ghana Health Service ethical review board and the Tamale Teaching hospital institutional review board for review. This trial would be registered with the appropriate clinical trial registry.

Some of the ethical considerations that will be strictly adhered to are as follows;

- All participants would receive the iron folic acid interventions
- Children randomized into no locally available dietary intervention groups would receive the LAD intervention at the end of the study.

The study will:

- ensure appropriate, safe, non-discriminatory participation of all stakeholders.
- promise to pay special attention to the needs of children and other vulnerable groups. Field Staff will be oriented on gender and child right issues as a critical consideration for the data collection exercise.
- respect the confidentiality and anonymity of the response from participants. And all field staff will be trained and supervised accordingly.
- solicit the informed consent of all respondents during the data collection phase through filling of Informed Consent Forms that clearly explains the purpose and the benefits of the study. All participants will be made to participate in this survey voluntarily.
- adopt a community entry procedure suitable to the traditional and cultural practices of the project communities.

**3.18 Data Collection and Monitoring of Field Work**

Data collection will be guided by the data collection management plan and quality control strategies. Once data collection tools are finalised, the study team will mobilise in readiness for the fieldwork and general data collection.

The field level quality control strategy will be defined and articulated in a field manual providing guidance to all field staff during data collection. This will include guidelines about how to engage respondents, the communication channels during fieldwork and ethical standards for data collection.

On the field, the Field Coordinator will meet the enumerators at the end of each day to summarize and evaluate data collected, identify gaps and define ways to fill them. As indicated earlier, the study will adopt electronic data collection system (Kobo or ODK Collect) for the questionnaire using Tablets. Data will on daily basis be downloaded for review and cleaning.

**3.19 Data Cleaning and Editing**

With the mobile based data collection, data is sent regularly (at least daily) to a server and from the server downloaded in a format (exportable to STATA) and scanned for any errors. On an on-going basis, the PI will support the quantitative enumerators to clean the data. A four-stage process will be adopted for ensuring the quality of the quantitative data, as follows:

- *Each Quantitative Interviewer would be required to edit and check for errors on all surveys conducted before submitting the tablets to the PI.*
- *It would be compulsory for the PI to scrutinise the Surveys on the Tablets before submitting the day’s work onto the server.*
- *The PI (who has the overall responsibility of quality assurance) will download the data and scan for errors before approving the next schedule for the team.*
- *At the end of the study, the PI and data collection team will do a final review before the data is analysed.*

*S***3.20 Data Analysis**

Data analysis would be by intention to treat and per protocol basis. Descriptive statistics would be used to describe and display a baseline characteristics of study participants by study arms to allow for comparing of the study arm after randomisation. The primary outcomes are the change in mean haemoglobin concentration and change in proportion of iron deficiency calculated from serum iron results for each arm of the study. This would be obtained by computing the mean change in haemoglobin concentration of each study participant within the study arm. A two-sample Student’s t-test assuming equal variances would be performed to test the hypothesis that the resulting mean haemoglobin from the two arms of the study are equal. Change in proportion of anaemic (ie, haemoglobin concentration <110 g/L), severely anaemic (ie, haemoglobin concentration <70 g/L) would be obtained for all groups. Nutritional z scores will be calculated according to the WHO growth reference curves with the use of Epi Info version 7.2.2.2. Weight-for-height, height-for-age, and weight-for-age z scores of less than −2 would be classified as wasting, stunting, and underweight, respectively; z scores of less than −3 would be considered to indicate severe wasting, severe stunting, or severe underweight. Underweight (ie, weight-for-age Z scores less than −2), and wasted (ie, weight-for-height Z scores less than −2); mean prevalence of diarrhoea, dysentery, and acute respiratory infection based on 7-day maternal recall and cumulative mortality would be analysed.

The trial is designed with the sample size and power considerations to detect a 0·2 difference in length-for-age, height for weight Z scores, a reduction of 8 percentage points in stunting, and a 2·6 g/L shift in haemoglobin for the marginal effect of either intervention, with 90% power and type 1 error of 5%. Selected covariates would be entered in a multivariable regression model; a forward stepwise selection procedure would be implemented with p<0·2 to enter. A log-binomial specification would be used to facilitate estimation of Risk Ratios (RR).

Depending on the analysis, other methods for comparison of groups while accounting for within-cluster correlation would include multinomial and ordinal regression models with robust variance estimation.

In a per-protocol analysis, the study will examine the effect of the interventions when behaviour change modules were delivered at high fidelity (hereby predefined for the Locally available dietary intervention groups as receiving twelve core modules and for all study groups, receiving all iron folic acid as scheduled with a missed dose rate less done 5%, calculated positive change in weekly dietary audit of intake of promoted locally available dietary intervention.

**3.21 Participant flow**

Figure 3.2 displays the consolidated standards of reporting trials (CONSORT) flow diagram for this study. It will be populated after the trial.

The flow diagram for the study can be represented as shown in the figure below;

**Screening**

Assessed for eligibility (n=No of Household/clusters)

Excluded (n= number of households)

Not Meeting inclusion criteria (n=)

Decline to participate (n=)

Other reasons (n=)

Assessed for eligibility (n=No of Household (clusters))

**Enrolment**

**Iron + Folic Acid:**

**Allocated intervention**

**Cluster (n=)**

**Children (n=)**

**Did not receive**

**Cluster (n=)**

**Children (n=)**

**Iron, Folic Acid Albendazole:**

**Allocated intervention**

**Cluster (n=)**

**Children (n=)**

**Did not receive**

**Cluster (n=)**

**Children (n=)**

**Iron, Folic Acid LAD**

**Allocated intervention**

**Cluster (n=)**

**Children (n=)**

**Did not receive**

**Cluster (n=)**

**Children (n=)**

**Iron, Folic Acid Albendazole LAD**

**Allocated intervention**

**Cluster (n=)**

**Children (n=)**

**Did not receive**

**Cluster (n=)**

**Children (n=)**

**Allocation**

**Loss to follow-up**

**Clusters (n=)**

**Children (n=)**

**Discontinued treatment**

**Clusters (n=)**

**Children (n=)**

Three-month intervention

**Loss to follow-up**

**Clusters (n=)**

**Children (n=)**

**Discontinued treatment**

**Clusters (n=)**

**Children (n=)**

**Loss to follow-up**

**Clusters (n=)**

**Children (n=)**

**Discontinued treatment**

**Clusters (n=)**

**Children (n=)**

**Loss to follow-up**

**Clusters (n=)**

**Children (n=)**

**Discontinued treatment**

**Clusters (n=)**

**Children (n=)**

**Follow-up**

**Analysed**

**Clusters (n=)**

**Children (n=)**

**Excluded from analysis**

**Clusters (n=)**

**Children (n=)**

**Analysed**

**Clusters (n=)**

**Children (n=)**

**Excluded from analysis**

**Clusters (n=)**

**Children (n=)**

**Analysed**

**Clusters (n=)**

**Children (n=)**

**Excluded from analysis**

**Clusters (n=)**

**Children (n=)**

**Analysed**

**Clusters (n=)**

**Children (n=)**

**Excluded from analysis**

**Clusters (n=)**

**Children (n=)**

**Analysed**

Figure 3.2 Consolidated standards of reporting trials (CONSORT) flow diagram for this study

**3.22 Work Plan**

The work plan is prepared based on the proposed methodology is as shown in table 3.3

**Table 3.3: work plan**

|  |  | **Period (Months)** | | | | | | |
| --- | --- | --- | --- | --- | --- | --- | --- | --- |
| **No.** | **Activities** | **1** | **2** | **3** | **4** | **5** | **6** | **7** |
| **1.0** | **Start-up Activities and Document Review** |  |  |  |  |  |  |  |
| 1.1 | Final correction of study protocol |  |  |  |  |  |  |  |
| 1.2 | Desk Review |  |  |  |  |  |  |  |
| 1.3 | Reconnaissance activities |  |  |  |  |  |  |  |
| 1.4 | Development of Management Plan, Training |  |  |  |  |  |  |  |
| **2.0** | **Mobilisation and Preparatory Activities for Field** |  |  |  |  |  |  |  |
| 2.1 | Development of Survey Instruments and related Protocols |  |  |  |  |  |  |  |
| 2.2 | Recruitment of Enumerators |  |  |  |  |  |  |  |
| 2.3 | Training of Enumerators |  |  |  |  |  |  |  |
| 2.4 | Pre-testing of Tools and Skills (Quantitative and Qualitative) |  |  |  |  |  |  |  |
| 2.5 | Finalisation of Tools (and upload on survey platform) and Printing of other instruments and manuals |  |  |  |  |  |  |  |
| 2.6 | Mobilisation/Procurement of Logistics and Equipment |  |  |  |  |  |  |  |
| **3.0** | **Data Collection and Data Cleaning** |  |  |  |  |  |  |  |
| 3.1 | Quantitative Data Collection and Supervision |  |  |  |  |  |  |  |
| 3.2 | Data Cleaning, Editing and Transcription |  |  |  |  |  |  |  |
| 3.3 | Interventions |  |  |  |  |  |  |  |
| **4.0** | **Data Analysis, Reporting and Debriefing** |  |  |  |  |  |  |  |
| 4.1 | Data cleaning and analysis |  |  |  |  |  |  |  |
| 4.2 | Drafting of Report |  |  |  |  |  |  |  |
| 4.3 | Review of First Draft with Comments from Supervisor |  |  |  |  |  |  |  |
| 4.5 | Presentation of Findings at journal club meeting |  |  |  |  |  |  |  |
| 4.6 | Finalised Draft Report with Comments from supervisors and colleagues |  |  |  |  |  |  |  |
| **5.0** | **Submission of Data Reports** |  |  |  |  |  |  |  |
| 5.1 | Submission of Final Report (Hard and Soft Copies) |  |  |  |  |  |  |  |

**3.23 Budget**

The budget for the project is summarised under the following headings: Summary of costing, Fee for staff, administrative and office, transportation and training, Laboratory reagent and equipment use and intervention cost. Table 3.4 displays the budget items.

**Table 3.4.1 Summary of Costing**

| **SUMMARY OF COSTING** |  |  |  |
| --- | --- | --- | --- |
| **COST ITEM** |  | **Rates (USD)** | **GHc** |
| FEES FOR STAFF | cost to be borne by PI | |  |
| ADMINISTRATIVE AND OFFICE COST | cost to be borne by PI | |  |
| TRANSPORTATION AND TRAINING |  | 1210.00 |  |
| LABORATORY REAGENT AND EQUIPMENT USE |  | 3065.00 | 15325 |
| INTERVENTION COST |  | 600.00 | 3000 |
| **TOTAL GROSS BUDGET** | Received funding | **4875.00** | **24375** |

**Table 3.4.2 Supplies**

| **SUPPLIES {LABORATORY REAGENT (base line and post intervention budget for 450 children. excess to take care of controls, calibrations and repeats)}** | | | |  |
| --- | --- | --- | --- | --- |
| **ITEM** | **No.** | **Number of tests** | **Rates (USD) per Each** | **Subtotal (USD)** |
| Full blood count reagents |  |  |  |  |
| Human count diluent | 1.5 | 450 | 76 | 114 |
| HC Lyse | 1.5 | 450 | 90 | 135 |
| HC cleaner | 1.5 | 450 | 64 | 96 |
| Control | 1 | 450 | 160 | 160 |
| Serum Ferritin |  | 450 | 0.9 | 405 |
| Total Iron Binding capacity |  | 450 | 0.9 | 405 |
| Serum Transferin |  | 450 | 0.9 | 405 |
| Serum Iron |  | 450 | 0.9 | 405 |
| Serum Vitamin B12 |  | 450 | 0.9 | 405 |
| Serum Folate |  | 450 | 0.9 | 405 |
| Normal saline | 2 L |  | 2 | 2 |
| Giemsa powder for malaria examination | 12.5 grams |  | 40 | 40 |
| Absolute methanol for malaria and film comment | 1L |  | 5 | 5 |
| Glycerol for malaria examination | 1 L |  | 5 | 5 |
| Leishman Powder for film comment | 12.5 grams |  | 40 | 40 |
| Microscope cover slides | 5 | 1.6 per 100 | 1.6 | 8 |
| microscope slides | 20 packs | 1.5 per pack of 50 | 1.5/50 | 30 |
| **Subtotal** |  |  |  | **3065** |

The minimum sample size of 184. However 200 was used for budgeting to take care of any broken, spilled out syrups that could occur in the administration of the drugs at home. The syrup requirement of an average two and half year old was used in the calculation of the number of bottles of iron and folic acid syrup required by each child.

**Table 3.4.3 Transportation and training**

| **TRANSPORATION AND TRAINING COST** | | |  |  |
| --- | --- | --- | --- | --- |
| **ITEM** | **No.** | **Number of days** | **Rates (USD) per day** | **Subtotal (USD)** |
| Vehicle Use | 1 | 13 | 50 | 650 |
| Fuel | 1 | 13 | 20 | 260 |
| Training Cost | 10 | 3 | 10 | 300 |
| **Subtotal** |  |  |  | **1210** |

| **INTERVENTION** |  |  |  |  |
| --- | --- | --- | --- | --- |
| **ITEM** | **No.** | Number | **Price (USD) per Each** | **Subtotal (USD)** |
| Iron folic acid syrup | 200 x 5 (200mls) | 1000 | 0.6 | 600 |
| **Subtotal** |  |  |  | **600** |

**Table 3.4.4 Intervention**

**Reference**

1. Vos T, Abajobir AA, Abate KH, Abbafati C, Abbas KM, Abd-Allah F, et al. Global, regional, and national incidence, prevalence, and years lived with disability for 328 diseases and injuries for 195 countries, 1990–2016: a systematic analysis for the Global Burden of Disease Study 2016. Lancet. 2017;390:1211–59. doi:10.1016/S0140-6736(17)32154-2.

2. Kassebaum NJ, Jasrasaria R, Naghavi M, Wulf SK, Johns N, Lozano R, et al. A systematic analysis of global anemia burden from 1990 to 2010. Blood. 2014;123:615–24. doi:10.1182/blood-2013-06-508325.

3. Pasricha S-R. Anemia: a comprehensive global estimate. Blood. 2014;123:611–2. doi:10.1182/blood-2013-12-543405.

4. Petry N, Olofin I, Hurrell RF, Boy E, Wirth JP, Moursi M, et al. The Proportion of Anemia Associated with Iron Deficiency in Low, Medium, and High Human Development Index Countries: A Systematic Analysis of National Surveys. Nutrients. 2016;8:693. doi:10.3390/nu8110693.

5. Organization WH. The global prevalence of anaemia in 2011. 2015.

6. Stevens GA, Finucane MM, De-Regil LM, Paciorek CJ, Flaxman SR, Branca F, et al. Global, regional, and national trends in haemoglobin concentration and prevalence of total and severe anaemia in children and pregnant and non-pregnant women for 1995–2011: a systematic analysis of population-representative data. Lancet Glob Heal. 2013;1:e16–25. doi:10.1016/S2214-109X(13)70001-9.

7. Ewusie JE, Ahiadeke C, Beyene J, Hamid JS. Prevalence of anemia among under-5 children in the Ghanaian population: estimates from the Ghana demographic and health survey. BMC Public Health. 2014;14:626. doi:10.1186/1471-2458-14-626.

8. Luo R, Yue A, Zhou H, Shi Y, Zhang L, Martorell R, et al. The effect of a micronutrient powder home fortification program on anemia and cognitive outcomes among young children in rural China: a cluster randomized trial. BMC Public Health. 2017;17:738. doi:10.1186/s12889-017-4755-0.

9. Griffiths JK, Kikafunda JK. Childhood Threats to Adult Cognition in Sub-Saharan Africa: Malaria, Anemia, Stunting, Enteric Enteropathy, and the Microbiome of Malnutrition. In: Brain Degeneration and Dementia in Sub-Saharan Africa. Springer, New York, NY; 2015. p. 75–87. https://link.springer.com/chapter/10.1007/978-1-4939-2456-1_7.

10. Collaborators GBDCM, Wang H, Coates MM, Coggeshall M, Dandona L, Fraser M, et al. Global, regional, national, and selected subnational levels of stillbirths, neonatal, infant, and under-5 mortality, 1980–2015: a systematic analysis for the Global Burden of Disease Study 2015. Lancet. 2016;388:1725–74. https://www.scopus.com/inward/record.uri?eid=2-s2.0-84994071373&doi=10.1016%2FS0140-6736%2816%2931575-6&partnerID=40&md5=b82d5d41478fee622f68081c450b8254.

11. Liu L, Oza S, Hogan D, Perin J, Rudan I, Lawn JE, et al. Global, regional, and national causes of child mortality in 2000–13, with projections to inform post-2015 priorities: an updated systematic analysis. Lancet. 2015;385:430–40. doi:10.1016/S0140-6736(14)61698-6.

12. Scott SP, Chen-Edinboro LP, Caulfield LE, Murray-Kolb LE. The Impact of Anemia on Child Mortality: An Updated Review. Nutrients. 2014;6:5915–32. doi:10.3390/nu6125915.

13. Kassebaum NJ. The Global Burden of Anemia. Hematol Clin. 2016;30:247–308. doi:10.1016/j.hoc.2015.11.002.

14. Lopez A, Cacoub P, Macdougall IC, Peyrin-Biroulet L. Iron deficiency anaemia. Lancet. 2016;387:907–16. doi:10.1016/S0140-6736(15)60865-0.

15. Schümann K, Solomons NW. Perspective: What Makes It So Difficult to Mitigate Worldwide Anemia Prevalence? Adv Nutr. 2017;8:401–8. doi:10.3945/an.116.013847.

16. Hotez PJ, Beaumier CM, Gillespie PM, Strych U, Hayward T, Bottazzi ME. Advancing a vaccine to prevent hookworm disease and anemia. Vaccine. 2016;34:3001–5. doi:10.1016/j.vaccine.2016.03.078.

17. Organization WH. Haemoglobin concentrations for the diagnosis of anaemia and assessment of severity. 2011.

18. (GSS) GSS, (GHS) GHS, International I. Ghana Demographic and Health Survey 2014. Rockville, Maryland, USA: GSS, GHS, and ICF International; 2015.

19. GSS, GHS, ICF. Ghana Maternal Health Survey 2017: Key Findings. Accra, Ghana; 2018. https://www.dhsprogram.com/publications/publication-SR251-Summary-Reports-Key-Findings.cfm.

20. Hurrell R, Egli I. Iron bioavailability and dietary reference values. Am J Clin Nutr. 2010;91:1461S-1467S. doi:10.3945/ajcn.2010.28674F.

21. Abbaspour N, Hurrell R, Kelishadi R. Review on iron and its importance for human health. J Res Med Sci. 2014;19:164–74. https://www.ncbi.nlm.nih.gov/pmc/articles/PMC3999603/.

22. Georgieff MK. Iron assessment to protect the developing brain. Am J Clin Nutr. 2017;106 suppl_6:1588S-1593S. doi:10.3945/ajcn.117.155846.

23. Paganini D, Zimmermann MB. The effects of iron fortification and supplementation on the gut microbiome and diarrhea in infants and children: a review. Am J Clin Nutr. 2017;106 suppl_6:1688S-1693S. doi:10.3945/ajcn.117.156067.

24. Camaschella C. Iron-deficiency anemia. N Engl J Med. 2015;372:1832–1843. internal-pdf://0.0.0.162/NEJMra1401038.html.

25. Lönnerdal B. Excess iron intake as a factor in growth, infections, and development of infants and young children. Am J Clin Nutr. 2017;106 suppl_6:1681S-1687S. doi:10.3945/ajcn.117.156042.

26. Organization WH. Nutritional anaemias: tools for effective prevention and control. 2017.

27. Nations FAO of the U. The State of Food Security and Nutrition in the World 2017: Building Resilience for Peace and Food Security. FAO; 2017.

28. da Silva Lopes K, Takemoto Y, Garcia-Casal MN, Ota E. Nutrition-specific interventions for preventing and controlling anaemia throughout the life cycle: an overview of systematic reviews. Cochrane Database Syst Rev. 2018. internal-pdf://0.0.3.91/full.html.

29. Ademola SA. Approach to anaemia diagnosis in developing countries: focus on aetiology and laboratory work-up. 2015.

30. Lee H, Chen Y-PP. Cell morphology based classification for red cells in blood smear images. Pattern Recognit Lett. 2014;49:155–161. internal-pdf://0.0.3.142/S0167865514001871.html.

31. Sharourou ASA, Hassan MA, Teclebrhan MB, Alsharif HM, Alhamad SA, Alsinani TS. Anemia: its Prevalence, Causes, and Management. Egypt J Hosp Med. 2018;70. internal-pdf://0.0.3.93/abstract.html.

32. Calis JCJ, Phiri KS, Faragher EB, Brabin BJ, Bates I, Cuevas LE, et al. Research Article (New England Journal of Medicine) Severe anemia in Malawian children. Malawi Med J. 2016;28:99–107. internal-pdf://0.0.3.147/146318.html.

33. Zere E, Kirigia JM, Duale S, Akazili J. Inequities in maternal and child health outcomes and interventions in Ghana. BMC Public Health. 2012;12:252. doi:10.1186/1471-2458-12-252.

34. Sezer RG, Bozaykut A, Akoglu HA, Özdemir GN. The Efficacy of Oral Vitamin B12 Replacement for Nutritional Vitamin B12 Deficiency. J Pediatr Hematol Oncol. 2018;40:e69. doi:10.1097/MPH.0000000000001037.

35. Castillo L. Heavy Metals and Health. Nova Science Publishers, Incorporated; 2016.

36. De-Regil LM, Suchdev PS, Vist GE, Walleser S, Peña-Rosas JP. Home fortification of foods with multiple micronutrient powders for health and nutrition in children under two years of age. Evidence-Based Child Heal A Cochrane Rev J. 2013;8:112–201. internal-pdf://0.0.3.154/ebch.html.

37. Brown JE. Nutrition through the life cycle. Cengage learning; 2016.

38. Wambach K, Riordan J. Breastfeeding and human lactation. Jones & Bartlett Learning; 2016. internal-pdf://0.0.3.159/books.html.

39. Stuart-Macadam P. Breastfeeding in prehistory. In: Breastfeeding. Routledge; 2017. p. 75–100. internal-pdf://0.0.3.161/9781315081984-3.html.

40. Maonga AR, Mahande MJ, Damian DJ, Msuya SE. Factors affecting exclusive breastfeeding among women in Muheza District Tanga northeastern Tanzania: a mixed method community based study. Matern Child Health J. 2016;20:77–87. internal-pdf://0.0.3.160/s10995-015-1805-z.html.

41. Organization WH. Strategies to prevent anemia: Recommendations from an Expert Group Consultation. New Delhi India. 2016.

42. Cibulskis RE, Alonso P, Aponte J, Aregawi M, Barrette A, Bergeron L, et al. Malaria: global progress 2000–2015 and future challenges. Infect Dis poverty. 2016;5:61. internal-pdf://0.0.3.240/s40249-016-0151-8.html.

43. Raouf S, Mpimbaza A, Kigozi R, Sserwanga A, Rubahika D, Katamba H, et al. Resurgence of malaria following discontinuation of indoor residual spraying of insecticide in a previously high transmission intensity area of Uganda. Clin Infect Dis. 2017.

44. Papanikolaou G, Pantopoulos K. Systemic iron homeostasis and erythropoiesis. IUBMB Life. 2017;69:399–413.

45. Ray S, Chandra J, Bhattacharjee J, Sharma S, Agarwala A. Determinants of nutritional anaemia in children less than five years age. Int J Contemp Pediatr. 2016;3:403–408. internal-pdf://0.0.3.151/256.html.

46. Scaglioni S, De Cosmi V, Ciappolino V, Parazzini F, Brambilla P, Agostoni C. Factors influencing children’s eating behaviours. Nutrients. 2018;10:706. internal-pdf://0.0.3.247/706.html.

47. Organization WH. The State of Food Security and Nutrition in the World 2018: Building climate resilience for food security and nutrition. Food & Agriculture Org.; 2018. internal-pdf://0.0.3.237/books.html.

48. Prado EL, Dewey KG. Nutrition and brain development in early life. Nutr Rev. 2014;72:267–84. doi:10.1111/nure.12102.

49. Andersson O, Lindquist B, Lindgren M, Stjernqvist K, Domellöf M, Hellström-Westas L. Effect of Delayed Cord Clamping on Neurodevelopment at 4 Years of Age: A Randomized Clinical Trial. JAMA Pediatr. 2015;169:631–8. doi:10.1001/jamapediatrics.2015.0358.

50. Christian P, Mullany LC, Hurley KM, Katz J, Black RE. Nutrition and maternal, neonatal, and child health. Semin Perinatol. 2015;39:361–72. doi:10.1053/j.semperi.2015.06.009.

51. Roganović J, Starinac K. Iron Deficiency Anemia in Children. Curr Top Anemia. 2018. doi:10.5772/intechopen.69774.

52. Chong A, Cohen I, Field E, Nakasone E, Torero M. Iron deficiency and schooling attainment in peru. Am Econ J Appl Econ. 2016;8:222–55. internal-pdf://0.0.3.181/articles.html.

53. Moschovis PP, Wiens MO, Arlington L, Antsygina O, Hayden D, Dzik W, et al. Individual, maternal and household risk factors for anaemia among young children in sub-Saharan Africa: a cross-sectional study. BMJ Open. 2018;8:e019654. doi:10.1136/bmjopen-2017-019654.

54. Vázquez-López MA, López-Ruzafa E, Ibáñez-Alcalde M, Martín-González M, Bonillo-Perales A, Lendínez-Molinos F. The usefulness of reticulocyte haemoglobin content, serum transferrin receptor and the sTfR-ferritin index to identify iron deficiency in healthy children aged 1–16 years. Eur J Pediatr. 2019;178:41–9. doi:10.1007/s00431-018-3257-0.

55. Harms K, Kaiser T. Beyond soluble transferrin receptor: Old challenges and new horizons. Best Pract Res Clin Endocrinol Metab. 2015;29:799–810. doi:10.1016/j.beem.2015.09.003.

56. Ganasen M, Togashi H, Takeda H, Asakura H, Tosha T, Yamashita K, et al. Structural basis for promotion of duodenal iron absorption by enteric ferric reductase with ascorbate. Commun Biol. 2018;1:120. internal-pdf://0.0.3.249/s42003-018-0121-8.html.

57. Hurrell RF. Efficacy and Safety of Iron Fortification. In: Food Fortification in a Globalized World. Elsevier; 2018. p. 195–212. internal-pdf://0.0.3.253/B9780128028612000201.html.

58. Alina VR, Carmen MC, Sevastita M, Andruţa M, Vlad M, Ramona S, et al. Food Fortification through Innovative Technologies. In: Food Engineering. IntechOpen; 2019. internal-pdf://0.0.4.1/food-fortification-through-innovative-technologies.html.

59. Moretti D. Plant-Based Diets and Iron Status. In: Vegetarian and Plant-Based Diets in Health and Disease Prevention. Elsevier; 2017. p. 715–727. internal-pdf://0.0.3.255/B9780128039687000393.html.

60. Beal T, Massiot E, Arsenault JE, Smith MR, Hijmans RJ. Global trends in dietary micronutrient supplies and estimated prevalence of inadequate intakes. PLoS One. 2017;12:e0175554. doi:10.1371/journal.pone.0175554.

61. Muñoz M, Gómez-Ramírez S, Besser M, Pavía J, Gomollón F, Liumbruno GM, et al. Current misconceptions in diagnosis and management of iron deficiency. Blood Transfus. 2017;15:422.

62. Gupta A. Interventional Strategies for Prevention of Nutritional Anemia. In: Gupta A, editor. Nutritional Anemia in Preschool Children. Singapore: Springer Singapore; 2017. p. 223–33. https://doi.org/10.1007/978-981-10-5178-4_14.

63. Yimam Y, Degarege A, Erko B. Effect of anthelminthic treatment on helminth infection and related anaemia among school-age children in northwestern Ethiopia. BMC Infect Dis. 2016;16:613. doi:10.1186/s12879-016-1956-6.

64. Molla E, Mamo H. Soil-transmitted helminth infections, anemia and undernutrition among schoolchildren in Yirgacheffee, South Ethiopia. BMC Res Notes. 2018;11:585. doi:10.1186/s13104-018-3679-9.

65. Gwetu TP, Chhagan MK, Taylor M, Kauchali S, Craib M. Anaemia control and the interpretation of biochemical tests for iron status in children. BMC Res Notes. 2017;10:163. doi:10.1186/s13104-017-2472-5.

66. Abraham D, Kaliappan SP, Walson JL, Ajjampur SSR. Intervention strategies to reduce the burden of soil-transmitted helminths in India. Indian J Med Res. 2018;147:533. internal-pdf://0.0.3.202/PMC6118140.html.

67. Lavadenz F, Schultz L, Matala T, Oyaka O, Calvo N, Qamruddin J. NTDs and Deworming Africa Initiative. 2018. internal-pdf://0.0.3.201/29692.html.

68. Montresor A, Trouleau W, Mupfasoni D, Bangert M, Joseph SA, Mikhailov A, et al. Preventive chemotherapy to control soil-transmitted helminthiasis averted more than 500 000 DALYs in 2015. Trans R Soc Trop Med Hyg. 2017;111:457–463. internal-pdf://0.0.3.205/pmc5808863.html.

69. Organization WH. Guideline: Preventive chemotherapy to control soil-transmitted helminth infections in at-risk population groups. World Health Organization; 2017.

70. Service GS. 2010 Population and Housing Census, District Analytical Report, Kumbungu District. Accra, Ghana; 2014.

71. Hemming K, Marsh J. A menu-driven facility for sample-size calculations in cluster randomized controlled trials. Stata J. 2013;13:114–35.

72. Kim J, Shin W. How to Do Random Allocation (Randomization). Clin Orthop Surg. 2014;6:103–9. doi:10.4055/cios.2014.6.1.103.

73. Caulfield LE, Bose A, Chandyo RK, Nesamvuni C, de Moraes ML, Turab A, et al. Infant Feeding Practices, Dietary Adequacy, and Micronutrient Status Measures in the MAL-ED Study. Clin Infect Dis. 2014;59 suppl_4:S248–54. doi:10.1093/cid/ciu421.

74. Coates J, Swindale A, Bilinsky P. Household Food Insecurity Access Scale (HFIAS) for measurement of food access: indicator guide. Washington, DC Food Nutr Tech Assist Proj Acad Educ Dev. 2007;:34.

75. Swindale A, Bilinsky P. Household dietary diversity score (HDDS) for measurement of household food access: indicator guide. Washington, DC Food Nutr Tech Assist Proj Acad Educ Dev. 2006.

76. Kennedy G, Ballard T, Dop MC. Guidelines for measuring household and individual dietary diversity. Food and Agriculture Organization of the United Nations; 2011.

**Appendices**

Appendix 1: Informed consent form

**Informed Consent form for household heads**

This Informed Consent Form is for men or women who are house hold-heads whose children under five years, we are inviting to participate in research on anemia in children under five. The title of our research project is **EFFECTS OF LOCALLY AVAILABLE DIETARY INTERVENTION, IN THE COMMUNITY BASED MANAGEMENT OF ANAEMIA IN CHILDREN UNDER FIVE: KUMBUNGU CLUSTER RANDOMIZED CONTROLLED TRIAL**

Name of Principal Investigator: **Dr. Benjamin Nuertey**

Name of Organization: **West African college of physicians/ community health department, university of Ghana school of public Health/ Korle-Bu teaching Hospital**

**This Informed Consent Form has two parts:**

- **Information Sheet (to share information about the research with you)**
- **Certificate of Consent (for signatures if you agree to take part)**

**You will be given a copy of the full Informed Consent Form**

**PART I: Information Sheet**

**Introduction**

I am ………………………………, working on the research project to reduce the burden of anaemia in children under five. We are doing research on anaemia in children less than five years, which is very common in this country. I am going to give you information and invite your household to be part of this research. You do not have to decide today whether or not you will participate in the research. Before you decide, you can talk to anyone you feel comfortable with about the research.

There may be some words that you do not understand. Please ask me to stop as we go through the information and I will take time to explain. If you have questions later, you can ask them of me, the study doctor or the staff.

**Purpose of the research**

Anaemia in children under five years is one of the most common and dangerous diseases in this region. There are however ways to treat anaemia including medicine and changes to the food we eat. The drugs that are currently used to help people with anaemia are not as good as we would like them to be. In fact, many children continue to die every day because of anaemia. There are new ways of managing anaemia which may work better. The reason we are doing this research is to find out if the new ways to manage anaemia are better than drug drugs alone which is currently being used.

**Type of Research Intervention**

This research is cluster randomized control trial. It will involve a daily intake of anaemia medication and six follow-up visits to your home. Community health nurses will use 15 minutes to talk to you when they visit your home. The study also involve a counselling approach which is a new way of improving anaemia.

**Participant selection**

We are selecting all households with children under five years to participate in the research on the new ways of managing anaemia

**Voluntary Participation**

Your participation in this research is entirely voluntary. It is your choice whether to participate or not. Whether you choose to participate or not, all you can visit any clinic of your choice to manage your child should the need arise and nothing will change. You may change your mind later and stop participating even if you agreed earlier

**Procedures and Protocol**

In this template, this section has been divided into two: firstly, an explanation of unfamiliar procedures and, secondly, a description of process.

**A. Unfamiliar Procedures**

1. Because we do not know if the new method is better than the currently available drug for treating anaemia alone, we need to compare the two. To do this, we will put people taking part in this research into two groups. The groups are selected by chance, as if by tossing a coin.
2. Participants in one group will be given the new method and the currently available drugs for treating anaemia while participants in the other group will be given the drug that is currently being used for anaemia. It is important that neither you nor we know which of the two drugs you are given except the nurse who would visit your home and give you the new treatment. This information will be in our files, but we will not look at these files until after the research is finished. This is the best way we have for testing without being influenced by what we think or hope might happen. We will then compare which of the two has the best results.
3. The healthcare workers will be looking after you and the other participants very carefully during the study. If we are concerned about what the drug is doing, we will find out which drug you are getting and make changes. If there is anything you are concerned about or that is bothering you about the research please talk to me or one of the other researchers
4. You will receive the treatment of your condition according to national guidelines. This means that your child will be examined and laboratory test carried free for us to confirm the cause of your child’s anaemia
5. We will take blood from your child’s arm using a syringe and needle. Each time we will take about this much blood (a small spoon) in at the beginning and after three months. At the end of the research, in 1 year, any leftover blood sample will be destroyed.

**B. Description of the Process**

During the research we may make six visits to your home.

- In the first visit, a small amount of blood, equal to about a teaspoon, will be taken from your child’s arm with a syringe. This blood will be tested anaemia and other things that would help us know the cause of the anaemia. We would also take urine and stool sample from your child. We will also ask you a few questions about your child’s general health and measure how tall you are and how much your child weigh.
- At the next visits, which will be two weekly, you will again be asked some questions about your health and then you will be given medicine used for the treatment of anaemia as explained before
- After three months, we will come back to your home again for a blood test. This will involve tested anaemia and other tests that would help us know the cause of the anaemia. We would also take urine and stool sample from your child.

**Duration**

The research’s field work takes place over 3 months in total.

**Side Effects**

As already mentioned, the drugs for treatment of anaemia can have some unwanted effects. It can make your child run diarrhoea and some abdominal upsets. It is possible that it may also cause some problems that we are not aware of. However, we will follow you closely and keep track of any unwanted effects or any problems. We may use some other medicines to decrease the symptoms of the side effects or reactions. Or we may stop the use of one or more drugs. If this is necessary we will discuss it together with you and you will always be consulted before we move to the next step.

**Risks**

By participating in this research, it is possible that you will be at greater risk than you would otherwise be. There is, for example, a risk that your child’s disease will not get better and that the new medicine doesn't work even as well as the old one. While the possibility of this happening is very low, you should still be aware of the possibility. We will try to decrease the chances of this event occurring, but if something unexpected happens, we will provide you with the necessary care

**Benefits**

If you participate in this research, you will have the following benefits: any interim illnesses will be treated at no charge to you. If your child falls sick during this period he/she will be treated free of charge. There may not be any benefit for you but your child’s participation is likely to help us find the answer to the research question. There may not be any benefit to the society at this stage of the research, but future generations are likely to benefit.

**Reimbursements**

You will not be given any money or gifts to take part in this research.

**Confidentiality**

With this research, something out of the ordinary is being done in your community. It is possible that if others in the community are aware that you are participating, they may ask you questions. We will not be sharing the identity of those participating in the research.

The information that we collect from this research project will be kept confidential. Information about you that will be collected during the research will be put away and no-one but the researchers will be able to see it. Any information about you will have a number on it instead of your name. Only the researchers will know what your number is and we will lock that information up with a lock and key. It will not be shared with or given to anyone.

**Sharing the Results**

The knowledge that we get from doing this research will be shared with you through community meetings before it is made widely available to the public. Confidential information will not be shared. There will be small meetings in the community and these will be announced. After these meetings, we will publish the results in order that other interested people may learn from our research

**Right to Refuse or Withdraw**

You do not have to take part in this research if you do not wish to do so. You may also stop participating in the research at any time you choose. It is your choice and all of your rights will still be respected

**Who to Contact**

If you have any questions you may ask them now or later, even after the study has started. If you wish to ask questions later, you may contact any of the following: [Dr. Benjamin Nuertey, Tamale Teaching Hospital/ telephone number 0246968106/e-mail; ben.nuertey@gmail.com]

**This proposal has been reviewed and approved by Ghana health service ethical review committee which is a committee whose task it is to make sure that research participants are protected from harm. If you wish to find about more about the IRB, contact: Ghana health service ethical review,** [**ghserc@gmail.com**](mailto:ghserc@gmail.com)**; 0302681109**

**PART II: Certificate of Consent**

I have read the foregoing information, or it has been read to me. I have had the opportunity to ask questions about it and any questions that I have asked have been answered to my satisfaction. I consent voluntarily to participate as a participant in this research.

**Print Name of household head__________________**

**Signature of household head ___________________**

**Date ___________________________**

**Day/month/year**

**If illiterate**

*A literate witness must sign (if possible, this person should be selected by the participant and should have no connection to the research team). Participants who are illiterate should include their thumb-print as well.*

**I have witnessed the accurate reading of the consent form to the potential participant, and the individual has had the opportunity to ask questions. I confirm that the individual has given consent freely.**

***Print name of witness_____________________ AND Thumb print of participant***

***Signature of witness ______________________***

**Date ________________________**

**Day/month/year**

**Statement by the researcher/person taking consent**

I have accurately read out the information sheet to the potential participant, and to the best of my ability made sure that the participant understands that the following will be done:

1. Blood, urine, stool samples and some measurements would be taken before and after 3 months of the study
2. Participation is voluntary, can stop at any time
3. We would follow up the household children in the study for 3 months

I confirm that the participant was given an opportunity to ask questions about the study, and all the questions asked by the participant have been answered correctly and to the best of my ability. I confirm that the individual has not been coerced into giving consent, and the consent has been given freely and voluntarily.

 A copy of this ICF has been provided to the participant.

Name of Researcher/person taking the consent________________________

Signature of Researcher /person taking the consent__________________________

Date ___________________________

Day/month/year

Appendix 2: Household socio-demographic questionnaire

Name of Household: …………………………………………………..household ID……..

Number in household……………………………………..

Number of rooms……………………………………………

Number of adults………………………………………………….

Number of Children less than 5 years………………………………………………

Number of children above 5 years ……………………………………………….

Occupation of family head……………………………………

Appendix 3: **Household Food Insecurity Access Scale (HFIAS) Measurement Tool**

| No | Question | Response | Code |
| --- | --- | --- | --- |
| 1 | In the past four weeks, did you worry that your household would not have enough food? | 0 = No (skip to Q2)  1=Yes | FS1 |
| 1a | How often did this happen? | 1 = Rarely (once or twice in the past four weeks)  2 = Sometimes (three to ten times in the past four weeks)  3 = Often (more than ten times in the past four weeks) | FS1a |
| 2 | In the past four weeks, were you or any household member not able to eat the kinds of foods you preferred because of a lack of resources? | 0 = No (skip to Q3)  1=Yes | FS2 |
| 2a | How often did this happen? | 1 = Rarely (once or twice in the past four weeks)  2 = Sometimes (three to ten times in the past four weeks)  3 = Often (more than ten times in the past four weeks) | FS2a |
| 3 | In the past four weeks, did you or any household member have to eat a limited variety of foods due to a lack of resources? | 0 = No (skip to Q4)  1 = Yes | FS3 |
| 3a | How often did this happen? | 1 = Rarely (once or twice in the past four weeks)  2 = Sometimes (three to ten times in the past four weeks)  3 = Often (more than ten times in the past four weeks) | FS3a |
| 4 | In the past four weeks, did you or any household member have to eat some foods that you really did not want to eat because of a lack of resources to obtain other types of food? | 0 = No (skip to Q5)  1 = Yes | FS4 |
| 4a | How often did this happen? | 1 = Rarely (once or twice in the past four weeks)  2 = Sometimes (three to ten times in the past four weeks)  3 = Often (more than ten times in the past four weeks) | FS4a |
| 5 | In the past four weeks, did you or any household member have to eat a smaller meal than you felt you needed because there was not enough food? | 0 = No (skip to Q6)  1 = Yes | FS5 |
| 5a | How often did this happen? | 1 = Rarely (once or twice in the past four weeks)  2 = Sometimes (three to ten times in the past four weeks)  3 = Often (more than ten times in the past four weeks) | FS5a |
| 6 | In the past four weeks, did you or any other household member have to eat fewer meals in a day because there was not enough food? | 0 = No (skip to Q7)  1 = Yes | FS6 |
| 6a | How often did this happen? | 1 = Rarely (once or twice in the past four weeks)  2 = Sometimes (three to ten times in the past four weeks)  3 = Often (more than ten times in the past four weeks) | FS6a |
| 7 | In the past four weeks, was there ever no food to eat of any kind in your household because of lack of resources to get food? | 0 = No (skip to Q8)  1 = Yes | FS7 |
| 7a | How often did this happen? | 1 = Rarely (once or twice in the past four weeks)  2 = Sometimes (three to ten times in the past four weeks)  3 = Often (more than ten times in the past four weeks) | FS7a |
| 8 | In the past four weeks, did you or any household member go to sleep at night hungry because there was not enough food? | 0 = No (skip to Q9)  1 = Yes | FS8 |
| 8a | How often did this happen? | 1 = Rarely (once or twice in the past four weeks)  2 = Sometimes (three to ten times in the past four weeks)  3 = Often (more than ten times in the past four weeks) | FS8a |
| 9 | In the past four weeks, did you or any household member go a whole day and night without eating anything because there was not enough food? | 0 = No (questionnaire is finished)  1 = Yes | FS9 |
| 9a | How often did this happen? | 1 = Rarely (once or twice in the past four weeks)  2 = Sometimes (three to ten times in the past four weeks)  3 = Often (more than ten times in the past four weeks) | FS9a |

Appendix 4: Household dietary diversity questionnaire

| ***DIETARY DIVERSITY QUESTIONNAIRE*** | | | | | | | | |
| --- | --- | --- | --- | --- | --- | --- | --- | --- |
| **Please describe the foods (meals and snacks) that you ate or drank yesterday during the day and night, whether at home or outside the home. Start with the first food or drink of the morning.** | | | | | | | | |
| *Write down all foods and drinks mentioned. When composite dishes are mentioned, ask for the list of ingredients*  *When the respondent has finished, probe for meals and snacks not mentioned.* | | | | | | | | |
| **Breakfast** | | **Snack** | **Lunch** | | **Snack** | **Dinner** | **Snack** | |
|  | |  |  | |  |  |  | |
| *When the respondent recall is complete, fill in the food groups based on the information recorded above. For any food groups not mentioned, ask the respondent if a food item from this group was consumed.* | | | | | | | | |
| **Question number** | **Food group** | | | **Examples** | | | | **YES=1 NO=0** |
| 1 | CEREALS | | | corn/maize, rice, wheat, sorghum, millet or any other grains or foods made from these (e.g. bread, noodles, porridge or other grain products) + *insert local foods e.g. porridge or pastes* | | | |  |
| 2 | WHITE ROOTS AND TUBERS | | | white potatoes, white yam, white cassava, or other foods made from roots | | | |  |
| 3 | VITAMIN A RICH VEGETABLES AND TUBERS | | | pumpkin, carrot, squash, or sweet potato that are orange inside + *other locally available vitamin A rich vegetables (e.g. red sweet pepper)* | | | |  |
| 4 | DARK GREEN LEAFY VEGETABLES | | | dark green/leafy vegetables, including wild forms + *locally available vitamin A rich leaves such as amaranth, cassava leaves, kale, spinach* | | | |  |
| 5 | OTHER VEGETABLES | | | other vegetables (e.g. tomato, onion, eggplant) + *other locally available vegetables* | | | |  |
| 6 | VITAMIN A RICH FRUITS | | | ripe mango, cantaloupe, apricot (fresh or dried), ripe papaya, dried peach, and 100% fruit juice made from these + *other locally available vitamin A rich fruits* | | | |  |
| 7 | OTHER FRUITS | | | other fruits, including wild fruits and 100% fruit juice made from these | | | |  |
| 8 | ORGAN MEAT | | | liver, kidney, heart or other organ meats or blood-based foods | | | |  |
| 9 | FLESH MEATS | | | beef, pork, lamb, goat, rabbit, game, chicken, duck, other birds, insects | | | |  |
| 10 | EGGS | | | eggs from chicken, duck, guinea fowl or any other egg | | | |  |
| 11 | FISH AND SEAFOOD | | | fresh or dried fish or shellfish | | | |  |
| 12 | LEGUMES, NUTS AND SEEDS | | | dried beans, dried peas, lentils, nuts, seeds or foods made from these (eg. hummus, peanut butter) | | | |  |
| 13 | MILK AND MILK PRODUCTS | | | milk, cheese, yogurt or other milk products | | | |  |
| 14 | OILS AND FATS | | | oil, fats or butter added to food or used for cooking | | | |  |
| 15 | SWEETS | | | sugar, honey, sweetened soda or sweetened juice drinks, sugary foods such as chocolates, candies, cookies and cakes | | | |  |
| 16 | SPICES, CONDIMENTS, BEVERAGES | | | spices (black pepper, salt), condiments (soy sauce, hot sauce), coffee, tea, alcoholic beverages | | | |  |
| Individual level | Did you eat anything (meal or snack) OUTSIDE the home yesterday? | | | | | | |  |

Appendix 5: Child questionnaire/ data capture sheet

Child ID ………………………….

Household ID ………………………………

Age…………………………………. Date of Birth………………….

Sex …………………………………. Schooling: YES NO

Anthropometric: ……………………………………

Weight ………………..height/length ………………….Head circumference……………….

Recent illness:………………………………………………..
