# Supplementary material for: Effects of a locally available dietary interventions counselling on the community-based management of anaemia in children under five years in Ghana: Kumbungu cluster randomized controlled trial protocol
Source: PLoS One. 2022 Apr 21;17(4):e0266157. doi: 10.1371/journal.pone.0266157 (PMC9022816; doi:10.1371/journal.pone.0266157)
Supplement: S2 Text — (DOCX) [file pone.0266157.s002.docx]

**SPIRIT checklist**

| **Administrative information** | |
| --- | --- |
| 1. Title | **Effects of a locally available dietary interventions counselling on the community-based management of anaemia in children under five in Ghana: Kumbungu cluster randomized controlled trial protocol** |
| 1. Trial registration | Pan African Trial Registry ([www.pactr.org](http://www.pactr.org)) **PACTR201906918438423** |
| 1. Protocol Version | **Corrected version 2** |
| 1. Funding | Part funding for this project was received from the Ghana-Michigan Collaborative funding program, which awards administers the Hauslohner Award. The principal investigator for this study would fund the remaining amount. |
| 1. Roles and Responsibilities |  |
| 5A: Contribution | BDN conceived the study. BDN, AEY, JAA, RBB initiated the study design, drafted and reviewed the protocol. |
| 5B: Sponsor contact | **Trial Part sponsor**: Ghana-Michigan Collaborative funding program, University of Michigan  Sponsor’s reference: Hauslohner Award  Contact name: Cheryl A. Moyer, PhD, MPH  **Address:** University of Michigan Medical School  1111 Catherine St., 221 Victor Vaughn Building  Ann Arbor, MI 48109  **Telephone**: +1-734-615-2838  **Email**: [camoyer@umich.edu](mailto:skouassi@umich.edu) |
| 5C: Sponsor and Funder | This funding source had no role in the design of this study and will not have any role during its execution, analyses, interpretation of the data, or decision to submit results |
| 5D: Committees | **Principal Investigator and Research Physician:** Design and conduct of research, preparation of protocol, patient recruitment  Steering Committee: all other researchers, responsible for reviewing the progress of the study and necessary changes to the protocol to facilitate smooth running |
| **Introduction** | |
| 1. Background and Rationale |  |
| 6A: Background and Rationale | **Background:** Anaemia in children under five remains a significant cause of mortality and morbidity in low-middle income countries. Globally, 27% of the world’s population is anaemic of which developing countries account for more than 89% . The global prevalence is worse in Africa and Asia. Anaemia has the potential of maintaining the cycle of poverty as it prevents children from attaining their full development potential. Most of the causes of anaemia in children under-five are preventable. Locally available dietary (LAD) interventions may be the sustainable interventions to address the high prevalence of anaemia in our communities.  **Rationale:** The study seeks is to determine effective community approach at reducing the burden of anemia and it related complication in children.  The aim of the study is to determine the effect of counselling on Locally Available Diet, on anaemia among children aged 6 – 59 months in the Kumbungu District of the Northern Region, Ghana. |
| 6B: Choice of comparators | Children with anaemia in the control group will all receive iron and folic acid syrup which is the standard of care for patient with anemia |
| 1. Objectives | **Hypothesis:** there is no difference in the status of anaemia between the group on iron + folic acid + counselling on LAD and the group on iron + folic acid only. If any difference, it is by chance  **Primary Objective:** To test the effects of counselling on Locally Available Diet on anaemia among children aged 6-59 months in the Kumbungu district of the northern region, Ghana.  **Secondary Objectives:**   1. To determine the baseline prevalence of anaemia and iron deficiency anaemia among children under five years in the Kumbungu district of northern region. 2. To determine the factors associated with anaemia among children under five years in the Kumbungu district of Ghana |
| 1. Trial design | This study will be a community-based cluster randomized, controlled trial, with two parallel arms. The counselling on locally available diets would be administered at baseline and a month after. Primary outcome data would be collected at baseline (pre-intervention) and then at the end of 12 weeks. |
| **Methods: Participants, Interventions, outcomes** | |
| 1. Study Settings | The study would be conducted in the Kumbungu district of the Northern region. The northern region was selected because, it has one of the highest prevalence of anaemia in children under five. Several activities by Governmental and Non-Governmental Organizations (NGOs) at addressing the problem of anaemia have shown little improvement. The Northern region has 26 districts. The Kumbungu district has been chosen for the study because of its high prevalence of anaemia in children under five |
| 1. Eligibility Criteria | **Inclusion criteria**  ***Inclusion criteria at household level***  At the household level, household inclusion to the study must satisfy the following:   1. The household must have at least a child aged 6 to 56 months.   ***Inclusion criteria at the participant level;***   1. All children within the eligible household aged 6 to 56 months at baseline would be included to answer specific objectives one and two. 2. For specific objective three, only children with haemoglobin concentration measured at baseline less than 11.0 g/dl would be eligible for inclusion. 3. The child should be residing in the selected community for at least the past three month. 4. Must have a legal guardian capable of providing informed consent.   **Exclusion criteria**  Eligible children with any of the following would be excluded from the study:   1. Current infective illness (example; respiratory infection, diarrhoea) with fever. Current infective illness would be assessed from self-reported and verification of available medical records. 2. Diagnosed case of any clinical haemoglobinopathy (eg, beta-thalassemia major, HbE-beta thalassemia, Sickle cell disease). This will be assessed based on self-report and available medical and laboratory records. 3. Received iron supplements or iron-containing MMP in the previous month. |
| 1. Interventions |  |
| 11A: Interventions | Intervention pertains to selected intervention communities level. The clusters assigned to counselling on LAD at a household level would each receive targeted counselling on LAD intervention aimed at promoting the intake of locally available iron, folate and vitamin B12 rich foods, food rich in enhancers of iron absorption and discouraging the intake of food rich in inhibitors of iron absorption. The counselling would be administered at baseline and repeated monthly. Each session of counselling would last minimum of 20 minutes. The counselling guide is attached as appendix 8. The rest of the interventions are targeted at the individual participant level. All study participants would receive the current practiced standard treatment for anaemia; iron + folic acid. |
| 11B: Modifications | Syrup iron and folic acid may be given in two equally divided doses to improve gastrointestinal tolerance. |
| 11C: Adherence | Bi-weekly visit by community health nurses/ village health volunteer to ensure adherence to iron and folic acid syrup. |
| 11D: Concomitant care | nil |
| 1. Outcomes | **Primary Outcome:** The primary outcome measures are mean haemoglobin levels in study arms. The haemoglobin level would be measured using a haematological analyser that measures the full (complete) blood count. Haemoglobin would be measured at baseline and end of intervention. Mean haemoglobin concentration (Hb g/dL) and status of anaemia in children: Hb <11.0 g/dL; mild anaemia (10.0 g/dL ≥ Hb ≤ 10.9 g/dL), moderate anaemia (7.0 g/dL ≥ Hb ≤ 9.9 g/dL), and severe anaemia as Hb < 7.0 g/dl would be determined at baseline and at end of intervention  **Secondary outcome:** A number of secondary outcomes would be measured in this study. Secondary outcomes would include measurement of anthropometry, and dietary recall, serum iron, ferritin, and other parameters of full blood count. |
| 1. Participant timeline | \| **Primary and Secondary outcomes** \| \| \| **Baseline** \| **Week** \| \| **End-line** \| \| --- \| --- \| --- \| --- \| --- \| --- \| --- \| \| **Week 0** \| **4** \| **8** \| **Week 12** \| \| **Primary outcome** \| \| \|  \|  \|  \|  \| \| 1 \| \| Haemoglobin concentration \| X \|  \|  \| X \| \| **Secondary Outcomes** \| \| \|  \|  \|  \|  \| \| **2** \| **Anthropometry** \| \|  \|  \|  \|  \| \|  \| Weight \| \| X \|  \|  \| X \| \|  \| Length/Height \| \| X \|  \|  \| X \| \|  \| Mid Upper Arm Circumference \| \| X \|  \|  \| X \| \| **3** \| **Biochemical outcome** \| \|  \|  \|  \|  \| \|  \| Serum Ferritin \| \| X \|  \|  \| X \| \|  \| Serum Iron concentration \| \| X \|  \|  \| X \| \|  \| Serum transferrin saturation \| \| X \|  \|  \| X \| \|  \| Serum Vitamin B12 level \| \| X \|  \|  \| X \| \|  \| Serum folate \| \| X \|  \|  \| X \| \|  \|  \| \|  \|  \|  \|  \| \| **4** \| **Clinical Outcome** \| \|  \|  \|  \|  \| \|  \| ***General Examination*** \| \|  \|  \|  \|  \| \|  \| Oedema \| \| X \|  \|  \| X \| \|  \| Jaundice \| \| X \|  \|  \| X \| \|  \| Pallor \| \| X \|  \|  \| X \| \|  \| Skin and hair changes \| \| X \|  \|  \| X \| \|  \|  \| \|  \|  \|  \|  \| \| **5** \| **Dietary recall and quantification** \| \| \|  \|  \|  \| \|  \| 24 hour repeated food record \| \| X \|  \|  \| X \| \|  \| Enhancers of iron in diet \| \| X \| X \| X \| X \| \|  \| Inhibitors of iron in diet \| \| X \| X \| X \| X \| |
| 1. Sample Size | The sample size was calculated to determine the minimum difference in outcome of management of anaemia between arms of the study. The following information was used:   1. Estimated prevalence of anaemia at baseline is 82% from previous study [7], and an estimated 20% reduction after 12 weeks. 2. Estimated average household size; which is the average number of children per household is 1.7 (≈2) obtained by the ratio of number of children aged less than five years (7,101) to the number of households in the district (4,133). 3. An estimated intra-cluster correlation (ICC) of 0.010 4. Estimate of coefficient of variation of cluster sizes of 0.90 5. Power of at least 80% (0.80) 6. Significance level of 5% (0.05) 7. Allowing for a cluster randomization design effect of 1.03 and 8. A dropout rate of about 5% for the 12 weeks period, 9. A contamination effect of 10%   With the above considerations, and using a menu driven facility for sample size calculation in cluster randomized controlled trial available as add-on in STATA (version 11.1, StataCorp, Special Edition, College Station, Texas 77845 USA), the minimum sample size and number of household per arm was calculated. The minimum sample size per arm is 92 and the number of clusters per study arm is 54 households (minimum sample size/ average cluster size= 92/1.7). Considering this study proposes two parallel arms, the total minimum study sample size is 184 and the minimum number of households is 108 households. |
| 1. Recruitment | The process of approaching and enlisting households will be based on the following guidelines:   1. Conduct a quick House and Household Listing in all four selected communities 2. Survey eligible households with the selected village 3. Interview only Household Heads/Spouse (Care givers) per each household 4. All eligible children per selected household would be enumerated and included in the study |
| **Methods: assignment of interventions** | |
| 1. Allocation | Allocation at cluster level |
| 1. Blinding | The intervention is a counselling intervention which makes concealment and blinding a near impossible task. This is because, at the individual participant level, the mother/ care giver of the child should be aware that she/he is being counselled on some locally available diet interventions to improve anaemia in his/her child.  However an assessor blind to treatment allocation will conduct assessments regarding clinical recovery. |
| **Methods: Data collection, management, analysis** | |
| 1. Data collection Methods | The study will collect demographic and baseline functional information from the patient’s legally authorized representative and/or caregivers  Blood sample would be collected for assessment at baseline and endline  Clinical examination by a physician would be carried at baseline and endline |
| 1. Data Management | The data entry screens will resemble the paper forms. Data integrity will be enforced through a variety of mechanisms. Referential data rules, valid values, range checks, and consistency checks against data already stored in the database |
| 1. Statistical Methods | Data analysis would be by intention to treat and per protocol basis. Descriptive statistics would be used to describe and display a baseline characteristics of study participants by study arms to allow for comparing of the study arm after randomisation. The primary outcomes are the change in mean haemoglobin concentration and change in proportion of iron deficiency calculated from serum iron results for each arm of the study. This would be obtained by computing the mean change in haemoglobin concentration of each study participant within the study arm. Paired T test would be used to test the within groups mean change in haemoglobin before and after the intervention. Analysis of variance (equality of variance test statistic) would be used to test the mean haemoglobin change between the groups. Also, two sample test of proportions would be used to test the proportion with anaemia within and between the groups before and after the intervention. Change in proportion of anaemic (ie, haemoglobin concentration <110 g/L), severely anaemic (ie, haemoglobin concentration <70 g/L) would be obtained for all groups. Nutritional z scores will be calculated according to the WHO growth reference curves with the use of Epi Info version 7.2.2.2. Weight-for-height, height-for-age, and weight-for-age z scores of less than −2 would be classified as wasting, stunting, and underweight, respectively; z scores of less than −3 would be considered to indicate severe wasting, or severe underweight. Underweight (ie, weight-for-age Z scores less than −2), and wasting (ie, weight-for-height Z scores less than −2) would be analysed.  The trial is designed with the sample size and power considerations to detect a 0·2 difference in length-for-age, height for weight Z scores, a reduction of 8 percentage points in stunting, and a 2·6 g/L shift in haemoglobin for the marginal effect of either intervention, with 90% power and type 1 error of 5%. Selected covariates would be entered in a multivariable regression model; a forward stepwise selection procedure would be implemented with p<0·2 to enter. A log-binomial specification would be used to facilitate estimation of Risk Ratios (RR).  Depending on the analysis, other methods for comparison of groups while accounting for within-cluster correlation would include multinomial and ordinal regression models with robust variance estimation. |
| **Methods: monitoring** | |
| 1. Data monitoring | A short trial of 12 weeks duration and the intervention relates to a public health counseling on use of Locally available diet. As such, there would be no formal data monitoring committee. |
| 1. Harms | In this study an adverse event will be defined as any untoward medical occurrence in a child. All adverse events in the study will be recorded. An adverse event that meets the criteria for a serious adverse event will be reported to the local IRB |
| 1. Auditing | - |
| **Ethics and dissemination** | |
| 1. Research Ethics approval | Ethical clearance was obtained from the Tamale Teaching hospital institutional review board; ID No: TTHERC/20/06/29/01 |
| 1. Protocol Amendment | Any modifications to the protocol which may impact on the conduct of the study, potential benefit of the patient or may affect patient safety, including changes of study objectives, study design, patient population, sample sizes, study procedures, or significant administrative aspects will require a formal amendment to the protocol |
| 1. Consent or assent | Research community health nurses will obtain written consent from household heads willing to participate in the trial. |
| 1. Confidentiality | All study-related information will be stored securely at the study site. All participant information will be stored in locked file cabinets in areas with limited access. All laboratory specimens, reports, data collection, process, and administrative forms will be identified by a coded ID |
| 1. Declaration of interest | No competing interests |
| 1. Access to Data | All trial investigators would have access to data sets |
| 1. Ancillary and post-trial care | nil |
| 1. Dissemination Policy | All study results would be communicated to village or community leaders. |
| **Appendices** | |
| 1. Informed consent materials | Informed consent form is attached |
| 1. Biological specimens | - |
