# Supplementary material for: Effects of a locally available dietary interventions counselling on the community-based management of anaemia in children under five years in Ghana: Kumbungu cluster randomized controlled trial protocol
Source: PLoS One. 2022 Apr 21;17(4):e0266157. doi: 10.1371/journal.pone.0266157 (PMC9022816; doi:10.1371/journal.pone.0266157)
Supplement: S1 File — (DOCX) [file pone.0266157.s003.docx]

**Supplement 1: Household socio-demographic questionnaire**

Name of Household: …………………………………………………..household ID……..

Number in household……………………………………..

Number of rooms……………………………………………

Number of adults………………………………………………….

Number of Children less than 5 years………………………………………………

Number of children above 5 years ……………………………………………….

Number of Eligible children in household…………………………….

Occupation of family head……………………………………

Mother’s highest educational status……………………………..

Religion (mother)…………………………………..

**Supplement 2: Household Food Insecurity Access Scale (HFIAS) Measurement Tool**

| No | Question | Response | Code |
| --- | --- | --- | --- |
| 1 | In the past four weeks, did you worry that your household would not have enough food? | 0 = No (skip to Q2)  1=Yes | FS1 |
| 1a | How often did this happen? | 1 = Rarely (once or twice in the past four weeks)  2 = Sometimes (three to ten times in the past four weeks)  3 = Often (more than ten times in the past four weeks) | FS1a |
| 2 | In the past four weeks, were you or any household member not able to eat the kinds of foods you preferred because of a lack of resources? | 0 = No (skip to Q3)  1=Yes | FS2 |
| 2a | How often did this happen? | 1 = Rarely (once or twice in the past four weeks)  2 = Sometimes (three to ten times in the past four weeks)  3 = Often (more than ten times in the past four weeks) | FS2a |
| 3 | In the past four weeks, did you or any household member have to eat a limited variety of foods due to a lack of resources? | 0 = No (skip to Q4)  1 = Yes | FS3 |
| 3a | How often did this happen? | 1 = Rarely (once or twice in the past four weeks)  2 = Sometimes (three to ten times in the past four weeks)  3 = Often (more than ten times in the past four weeks) | FS3a |
| 4 | In the past four weeks, did you or any household member have to eat some foods that you really did not want to eat because of a lack of resources to obtain other types of food? | 0 = No (skip to Q5)  1 = Yes | FS4 |
| 4a | How often did this happen? | 1 = Rarely (once or twice in the past four weeks)  2 = Sometimes (three to ten times in the past four weeks)  3 = Often (more than ten times in the past four weeks) | FS4a |
| 5 | In the past four weeks, did you or any household member have to eat a smaller meal than you felt you needed because there was not enough food? | 0 = No (skip to Q6)  1 = Yes | FS5 |
| 5a | How often did this happen? | 1 = Rarely (once or twice in the past four weeks)  2 = Sometimes (three to ten times in the past four weeks)  3 = Often (more than ten times in the past four weeks) | FS5a |
| 6 | In the past four weeks, did you or any other household member have to eat fewer meals in a day because there was not enough food? | 0 = No (skip to Q7)  1 = Yes | FS6 |
| 6a | How often did this happen? | 1 = Rarely (once or twice in the past four weeks)  2 = Sometimes (three to ten times in the past four weeks)  3 = Often (more than ten times in the past four weeks) | FS6a |
| 7 | In the past four weeks, was there ever no food to eat of any kind in your household because of lack of resources to get food? | 0 = No (skip to Q8)  1 = Yes | FS7 |
| 7a | How often did this happen? | 1 = Rarely (once or twice in the past four weeks)  2 = Sometimes (three to ten times in the past four weeks)  3 = Often (more than ten times in the past four weeks) | FS7a |
| 8 | In the past four weeks, did you or any household member go to sleep at night hungry because there was not enough food? | 0 = No (skip to Q9)  1 = Yes | FS8 |
| 8a | How often did this happen? | 1 = Rarely (once or twice in the past four weeks)  2 = Sometimes (three to ten times in the past four weeks)  3 = Often (more than ten times in the past four weeks) | FS8a |
| 9 | In the past four weeks, did you or any household member go a whole day and night without eating anything because there was not enough food? | 0 = No (questionnaire is finished)  1 = Yes | FS9 |
| 9a | How often did this happen? | 1 = Rarely (once or twice in the past four weeks)  2 = Sometimes (three to ten times in the past four weeks)  3 = Often (more than ten times in the past four weeks) | FS9a |

**Supplement 3: Household dietary diversity questionnaire**

| ***DIETARY DIVERSITY QUESTIONNAIRE*** | | | | | | | | |
| --- | --- | --- | --- | --- | --- | --- | --- | --- |
| **Please describe the foods (meals and snacks) that you ate or drank yesterday during the day and night, whether at home or outside the home. Start with the first food or drink of the morning.** | | | | | | | | |
| *Write down all foods and drinks mentioned. When composite dishes are mentioned, ask for the list of ingredients*  *When the respondent has finished, probe for meals and snacks not mentioned.* | | | | | | | | |
| **Breakfast** | | **Snack** | **Lunch** | | **Snack** | **Dinner** | **Snack** | |
|  | |  |  | |  |  |  | |
| *When the respondent recall is complete, fill in the food groups based on the information recorded above. For any food groups not mentioned, ask the respondent if a food item from this group was consumed.* | | | | | | | | |
| **Question number** | **Food group** | | | **Examples** | | | | **YES=1 NO=0** |
| 1 | CEREALS | | | corn/maize, rice, wheat, sorghum, millet or any other grains or foods made from these (e.g. bread, noodles, porridge or other grain products) + *insert local foods e.g. porridge or pastes* | | | |  |
| 2 | WHITE ROOTS AND TUBERS | | | white potatoes, white yam, white cassava, or other foods made from roots | | | |  |
| 3 | VITAMIN A RICH VEGETABLES AND TUBERS | | | pumpkin, carrot, squash, or sweet potato that are orange inside + *other locally available vitamin A rich vegetables (e.g. red sweet pepper)* | | | |  |
| 4 | DARK GREEN LEAFY VEGETABLES | | | dark green/leafy vegetables, including wild forms + *locally available vitamin A rich leaves such as amaranth, cassava leaves, kale, spinach* | | | |  |
| 5 | OTHER VEGETABLES | | | other vegetables (e.g. tomato, onion, eggplant) + *other locally available vegetables* | | | |  |
| 6 | VITAMIN A RICH FRUITS | | | ripe mango, cantaloupe, apricot (fresh or dried), ripe papaya, dried peach, and 100% fruit juice made from these + *other locally available vitamin A rich fruits* | | | |  |
| 7 | OTHER FRUITS | | | other fruits, including wild fruits and 100% fruit juice made from these | | | |  |
| 8 | ORGAN MEAT | | | liver, kidney, heart or other organ meats or blood-based foods | | | |  |
| 9 | FLESH MEATS | | | beef, pork, lamb, goat, rabbit, game, chicken, duck, other birds, insects | | | |  |
| 10 | EGGS | | | eggs from chicken, duck, guinea fowl or any other egg | | | |  |
| 11 | FISH AND SEAFOOD | | | fresh or dried fish or shellfish | | | |  |
| 12 | LEGUMES, NUTS AND SEEDS | | | dried beans, dried peas, lentils, nuts, seeds or foods made from these (eg. hummus, peanut butter) | | | |  |
| 13 | MILK AND MILK PRODUCTS | | | milk, cheese, yogurt or other milk products | | | |  |
| 14 | OILS AND FATS | | | oil, fats or butter added to food or used for cooking | | | |  |
| 15 | SWEETS | | | sugar, honey, sweetened soda or sweetened juice drinks, sugary foods such as chocolates, candies, cookies and cakes | | | |  |
| 16 | SPICES, CONDIMENTS, BEVERAGES | | | spices (black pepper, salt), condiments (soy sauce, hot sauce), coffee, tea, alcoholic beverages | | | |  |
| Individual level | Did you eat anything (meal or snack) OUTSIDE the home yesterday? | | | | | | |  |

**Supplement 4: Child questionnaire/ data capture sheet**

Child ID ………………………….

Household ID ………………………………

Age…………………………………. Date of Birth………………….

Sex …………………………………. Schooling: YES…… NO……

Anthropometric

Weight ………………..height/length ………………….Head circumference……………….

MUAC……………………

Clinical examination findings Oedema Pallor

Jaundice Skin and hair changes

Other findings………………………………………………….

Recent illness:………………………………………………..

Nutritional diagnosis…………………………………………

Disabilities……………………………………………………..

**Supplement 5: Kumbungu Cluster Randomized Controlled Trial: Counselling guide on locally available diets: For care givers of children under five with anaemia**

- **Increase the consumption of iron-rich foods**,
  - primarily animal-source foods such as meat (especially red meat of all kinds), poultry and fish, but also
  - locally available iron-rich plant sources such as legumes,
- **Increase the consumption of foods that are rich in folate and vitamin A/carotenoid**,
  - orange-fleshed fruits and vegetables (e.g. orange-fleshed sweet potatoes, carrots),
  - green leafy vegetables such as ‘Alefu’, ‘Ayoyo’, ‘Ademe’, “bra”, beans leaves, okro, baobab leaves, kontomire
  - Dairy products, eggs, liver and fish oils.
  - Legumes; beans of all kinds including Bambara, soy, cowpea, black eye beans, agushi, neri, bungu, sesamin, dawadawa, groundnuts and its products like “kulkuli” and “kulikuli” powder
- **Enhancers of iron absorption: Add fruits and vegetables that are rich in citric or ascorbic acid (e.g. citrus fruits) to the diet, to increase the absorption of non-haem iron.**
  - Vitamin C degrades with cooking, so consumption of uncooked (or lightly cooked) fruits and vegetables with high vitamin C content should be encouraged (assuming considerations of food hygiene and food safety are addressed).
  - Add locally available fruits and vegetables rich in vitamin C;
    - baobab powder (high iron, vitamin C) for drink, porridge, ice cream,
    - Tamarind incorporated in to drinks and porridges
    - Oranges, other fruits; shea fruits, dawadawa, mangoes
- **Identify and promote culturally appropriate and feasible methods of food processing and preparation,**
  - To improve bioavailability and absorption.
    - *Iron*: germination, fermentation and soaking may improve absorption.
    - *Vitamin A*: short cooking times and steaming rather than boiling will maintain pro-vitamin A activity
  - Preparation in a manner acceptable to children of the various age group; cooking methods that results in the meat being tender enough for the children to have it should be employed
    - Mashing up
- **Avoid combining known inhibitors of iron absorption with meals that are high in iron content;**
  - for example: separate tea and coffee drinking from meal times; consumption 1–2 hours later will not inhibit iron absorption;
  - Pica including white clay (shile)
  - consume dairy products (“Waagashi” milk, cheese and other foods made from milk) as a between meal snack, not at a meal time

**Supplement 6:**

**FOOD FREQUENCY QUESTIONNAIRE**

**Kumbungu Cluster Randomized Controlled Trial**

**To be completed at baseline and end-line**

**Baseline three session End line session**

**Please tick (√) which ever apply**

**Community…………………………… Household…………………**

**Name: …………………………………. Age……… Sex……………**

| **Food Items** | **Daily** | **Weekly** | **Monthly** | **Rarely** | **Never** |
| --- | --- | --- | --- | --- | --- |
| **Breastmilk** |  |  |  |  |  |
| **Iron rich food and enhancers of its absorption** | | | | | |
| Red meat of all kinds;  E.g. Beef, goat, mutton, etc |  |  |  |  |  |
| Poultry: guinea fowl, duck, chicken |  |  |  |  |  |
| Eggs |  |  |  |  |  |
| Fish; (smoked, grilled, steamed) |  |  |  |  |  |
| Green leafy vegetables E.g.  Alefu’, ‘Ayoyo’, ‘Ademe’ |  |  |  |  |  |
| Baobab powder |  |  |  |  |  |
| Tamarind |  |  |  |  |  |
| Citrus fruits; orange, lemon, etc |  |  |  |  |  |
| Liver |  |  |  |  |  |
| **Other foods** | | | | | |
| Tea or coffee |  |  |  |  |  |
| Dairy products; Waagashi, milk,  cheese |  |  |  |  |  |
| Vegetable proteins (beans,soy,  agushie) |  |  |  |  |  |
| Fresh fruits(orange, watermelon etc) |  |  |  |  |  |
| Food supplements (e.g. multivitamins) |  |  |  |  |  |

**Supplement 7:**

**REPEATED 24-HOUR RECALL (THREE DAY FOOD RECORD)**

**Kumbungu Cluster Randomized Controlled Trial**

**To be completed three times per child; two weekdays and one weekend per session**

**Baseline three session End line session**

**Please tick (√) which ever apply**

**Community…………………………… Household…………………**

**Name: …………………………………. Age……… Sex……………**

**Three day Food record**

**WEEKDAY 1 WEEKDAY 2 WEEKEND**

**Please tick (√) which ever apply**

| **MEAL** | **FOOD CONSUMED** | **HANDY MEASURE** | **QUANTITY (G)** | **NUTRIENT CONTENT** |
| --- | --- | --- | --- | --- |
| **Example:** | **Hausa koko**  **Cowbell milk**  **koose** | **Two soup ladles**  **2 tablespoonful**  **1 small piece** | **Please leave this column blank (To be calculated from handy measure)** | **Please leave this column blank ( to be calculated based on quantity and food composition tables)** |
| **Breakfast** |  |  |  |  |
| **Snack** |  |  |  |  |
| **Lunch** |  |  |  |  |
| **Snack** |  |  |  |  |
| **Supper** |  |  |  |  |
| **Snack** |  |  |  |  |

**Participant flow**

The flow diagram for the study can be represented as shown in the figure below;

**Screening**

Assessed for eligibility (n=No of Household)

Excluded (n= number of households)

Not Meeting inclusion criteria (n=)

Decline to participate (n=)

Other reasons (n=)

Assessed for eligibility (n=No of Household)

**Enrolment**

**Iron + Folic Acid:**

**Allocated intervention**

**Households (n=)**

**Children (n=)**

**Did not receive**

**Cluster (n=)**

**Children (n=)**

**Iron + Folic Acid + Counselling on LAD**

**Allocated intervention**

**Households (n=)**

**Children (n=)**

**Did not receive**

**Cluster (n=)**

**Children (n=)**

**Allocation**

**Loss to follow-up**

**Households (n=)**

**Children (n=)**

**Discontinued treatment**

**Households (n=)**

**Children (n=)**

**Three-month intervention**

**Loss to follow-up**

**Households (n=)**

**Children (n=)**

**Discontinued treatment**

**Households (n=)**

**Children (n=)**

**Follow-up**

**Analysed**

**Households (n=)**

**Children (n=)**

**Excluded from analysis**

**Households (n=)**

**Children (n=)**

**Analysed**

**Households (n=)**

**Children (n=)**

**Excluded from analysis**

**Households (n=)**

**Children (n=)**

**Analysed**
