# Supplementary material for: Effects of a locally available dietary interventions counselling on the community-based management of anaemia in children under five years in Ghana: Kumbungu cluster randomized controlled trial protocol
Source: PLoS One. 2022 Apr 21;17(4):e0266157. doi: 10.1371/journal.pone.0266157 (PMC9022816; doi:10.1371/journal.pone.0266157)
Supplement: Attachment — Submitted filename: Response to Reviewers.docx [file pone.0266157.s004.docx]

17 May, 2021

Dear Editor

**Response to Reviewers’ comment**

Thank you for the opportunity to submit a revision to this manuscript:

PONE-D-20-28388

Effects of a locally available dietary interventions counselling on the community-based management of anaemia in children under five in Ghana: Kumbungu cluster randomized controlled trial protocol

I am grateful for the inputs made by you and the reviewers, which greatly improved this manuscript.

Please find below the point-by-point response to reviewer comments.

**Comments by Academic Editor:**

1. Please ensure that your manuscript meets PLOS ONE's style requirements, including those for file naming.
2. We note that Figure(s) 1 in your submission contain map images which may be copyrighted …….. If you are unable to obtain permission from the original copyright holder to publish these figures under the CC BY 4.0 license or if the copyright holder’s requirements are incompatible with the CC BY 4.0 license, please either i) remove the figure or ii) supply a replacement figure that complies with the CC BY 4.0 license. Please check copyright information on all replacement figures and update the figure caption with source information. If applicable, please specify in the figure caption text when a figure is similar but not identical to the original image and is therefore for illustrative purposes only.
3. Please include a caption for figure 1

**Response:**

*Thank you, the figure has been removed*

4. We note you have included a table to which you do not refer in the text of your manuscript. Please ensure that you refer to Table 1 in your text; if accepted, production will need this reference to link the reader to the Table.

**Response**

*Please this has been carried out*

5. Please include captions for your Supporting Information files at the end of your manuscript, and update any in-text citations to match accordingly. Please see our Supporting Information guidelines for more information: http://journals.plos.org/plosone/s/supporting-information.

**Response**

*Please this has been carried out*

**Reviewer #1:**

The authors describe testing a promising intervention for the prevention and treatment of anemia in young children in Africa.

The study design appears sound and methods are generally adequately described.

English errors occur throughout the manuscript and the figures and tables contain alignment and formatting errors.

***Response***

Thank you, English Errors corrected and the figure and table alignment and formatting errors corrected

**Comments:**

Please describe the diet of the communities and whether the residents have access to the foods/beverages you plan to include in the counseling intervention. Is there published evidence that consumption of iron inhibitors and lack of consumption of iron enhancers is occurring in these communities?

**response**

Majority of the people in the Kumbungu district are farmers who cultivate small fragmented parcel of lands [22]. Cattles are the most valuable livestock that are rarely sold or slaughtered except for extreme shock such as crop failure or famine [22]. Also, livestock and poultry are often kept by most household which provides immediate cash to purchase food, pay for medical bills, source of protein rich diet and also given out as gift/ sacrifice [23]. Previous studies in the district have found high intake of phytates and tea which are known inhibitors of iron absorption [23]. Locally available diets that contain high amount of vitamin C such as tamarind, ripe shea fruit and baobab are seasonal and when available, are not expensive to buy.

**comment**

Are animal foods commonly consumed?

***Response***

The following has been included on the section on study site; “*Culturally, meet is reserved for festive occasion. Animal food; anyone who can afford animal food is a plus. Though livestock, poultry are kept by some on small scale, free range level, the eggs and meat are often sold for cash.”*

**comment**

How will nutrient/dietary data be analyzed? What databases are to be used?

***Response***

Using a handy measure conversion, handbook, the gram weight of the food items consumed would be obtained and entered into the West Africa Food Analysis software for the analysis of nutrient/dietary data. The software makes use of the West African Food composition table.

This has been included in the data analysis section.

**Comment**

Is the sample size adequate to detect significant differences in the secondary outcome measures of s ferritin and iron?

***Response***

Thank you for pointing this to us. The sample size has been recalculated and powered to detect secondary outcome measures of serum ferritin and iron. Eight community clusters would be included and a minimum of 200 households with a minimum of 396 children would be included in the study.

**comment**

Have these communities been exposed to supplemental iron and folate previously?

***Response***

Individual anaemic children admitted or managed at health centres and or hospital might have been given supplemental iron and folate. However, there is no program in Ghana for iron and folate supplementation for children under 5 years. This statement was included in the discussion section.

**Comment**

What instructions are provided to the caregivers regarding administration of the supplements (with meals or not; morning or evening)?

**Response**

Women would be advised to administer the supplement to children in the morning 1 to 2 hours before or after meals and not to be combined with meals.

**comment**

Explain the training for the LAD counseling.

***Response***

Thank you for pointing this out. The following has been included in the section on intervention; last paragraph.

*“Counselling on LAD would be carried out by trained nurses with at least a diploma degree in nursing. A training on the counselling guide would be carried out by a team of nutritionist and doctor. The training is expected to last six hours and would include lecture, simulation and practical discussions. Food groups, locally available foods, enhancers of iron, inhibitors of iron, effective counselling methods would be discussed.”*

**comment**

The consent form should describe the collection of dietary data.

***Response***

Thank you. It has been included.

**Reviewer #2:**

For the secondary outcomes, diet recall is often not accurate. A better approach would be to have the participants record food intake daily for more accuracy.

***Response***

*Thank you for emphasizing this. We would take a 24hour repeated food record. Two on weekdays and one on a weekend at baseline and repeated at the end of the study.*

**Comment**

Also information on supplements used during the study, if any, by the households should be included.

***Response***

*Thank you very much. Supplements used during the study if any is a potential confounder and information on that would be collected. This has been included in the table 1 and the preceding text on secondary outcomes to me measured*

**Reviewer #3:**

Abstract

- since the cluster is the unit of randomisation, indicate the total number of children, households and community clusters to be included.

***Response***

Thank you, this has been clearly stated now as can be found in the section under sample size calculation and the abstract and restated here as follows: “***The minimum number of children per arm is 198 and the number of community clusters is 8 (4 per study arm). Also, considering this study proposes two parallel arms, the total minimum study sample size of children under five with anaemia is 396 and the minimum total number of community clusters is 8 and a minimum of 25 households per community cluster, 100 households per study arm and 200 households need to be enrolled for the study”.***

**Comment**

- clarify which aspects of anthropometry will be measured.

**response**

Weight, height/length, mid upper arm circumference was specified in the abstract section now.

**Comment**

Introduction

- typo in second-last paragraph of page 2 - 'diarrhoea'

- in first paragraph on page 3, you mean 'children under five years [of age]' not 'children lower than five years'. Also, 'which are respectively projected...' instead of 'is respectively projected...'

- second-last line on page 3 - you probably mean 'distinctive' rather than 'peculiar'.

***Response***

Really appreciate the suggestions; all were accepted and the changes effected.

**comment**

Methods

- there's a potentially fundamental problem with the study design. In the design section, you refer to the communities as the clusters. So your effective sample size is two clusters per arm; however in the sample size calculation, you refer to the households as the clusters, and indicate that the number of clusters per study arm is 54. This needs to be clarified. Either the community is a higher-level unit of stratification (in the same way one might conduct stratified randomisation by country in a multi-country study), or if it is indeed the unit of clustering then the study design and sample size calculation needs to be thought through, as it is not appropriate if cluster randomisation is at the community level.

***Response***

Thank you very much for pointing this out to us. We worked out the whole sample size calculation and design section accepting your useful recommendation. Community clusters were used instead of household clusters and the rational explained in the design section. In all, eight community clusters were to be included in the study after re-calculating the sample size to accommodate your suggestion. The design and sample size sections have been reviewed to reflect the changes.

**Comment**

- a more appropriate analysis would involve a mixed effects generalised linear model of the endline outcome measures adjusted for baseline measures (for continuous outcomes only - analysis of covariance), with further adjustment for age, sex and any stratification factors. Mixed effects models, or other hierarchical models, appropriately adjust for the clustering in the expected data. The proposed approach involving a t-test is not appropriate as it doesn't adjust for the clustered design, and doesn't allow for adjustment for baseline continuous outcomes (which improves efficiency - for continuous outcomes only).

***Response***

We agree with reviewer and have reviewed our initial analysis plan as below;

An impact evaluation analysis would be adopted to establish the hypothetical impact of the intervention. Multilevel mixed-effects generalized linear model (MEGLM) would be adopted based on the design nature of the study. The assumption for using MEGLM would allow for conditional normally of random effects. Very helpful.
