# Supplementary material for: Effects of a locally available dietary interventions counselling on the community-based management of anaemia in children under five years in Ghana: Kumbungu cluster randomized controlled trial protocol
Source: PLoS One. 2022 Apr 21;17(4):e0266157. doi: 10.1371/journal.pone.0266157 (PMC9022816; doi:10.1371/journal.pone.0266157)
Supplement: Attachment — Submitted filename: response to reviewer comment.docx [file pone.0266157.s005.docx]

**Reviewer #1**:

**Comment**

*The authors have made most of the recommended revisions and the manuscript is improved.*

*English errors remain and need correction. "Years" should be included when describing ages of children over 5 and adults. Include articles (such as "the") when appropriate and check agreement of nouns and verbs for singular/plural. "Meet" should be spelled "meat."*

**Response**

Thank you very much for your thorough review which improved the manuscript. All the suggested changes have been carried out. Thank you.

**Reviewer #3:**

**Comment**

*Thank you for your clear and comprehensive responses to the previous round of reviews. There are a number of key aspects of the protocol which I think would still benefit from improvement*.

**Response**

We are deeply grateful for your suggestions, education, and support particular with the statistical aspect of this protocol. Your inputs were very valuable to us. Thank you.

**Comment**

*First, I would suggest that you revisit your sample size calculation, as this aspect of the study design is critical but doesn't seem correct in the current version of the protocol.*

*The key parameters of the sample size calculation are that the prevalence of anaemia is 82% and you hope to see an absolute 22% reduction over 12 weeks. You hope to have 80% power to detect this difference, at the 5% level of significance.*

*The cluster design aspects of the trial are inter-related. The cluster size and number of clusters to include are within your control. However, the ICC (ie. how similar the outcome is between individuals in the same community) is a natural phenomenon which you cannot vary. The design effect is the inflation factor by which you increase the basic sample size to account for the clustered design; it is function of the cluster size, number of clusters and ICC and does not need to be presented/calculated separately as you have done in (7) in your description of the calculation.*

*Given that the cluster size and number of clusters is what is within your control, these are the only aspects of the design that you can change. Currently you have decided that you'll recruit about 50 children per cluster - this is the cluster size. With this cluster size, assuming an ICC of 0.01, you will need 6 clusters per arm - so your current estimate of 4 per arm would not be sufficient.*

*If you are really only able to work with 4 communities in each arm, then you need to increase the cluster size i.e. the number of children you recruit in each cluster. With a cluster size of 100, all else remaining the same, you need 4 community clusters per arm, i.e. 8 community clusters total and 800 children total. If you anticipate any losses to follow-up, contamination/cross-over e.t.c. then you need to appropriately inflate the expected number of children per arm so that your actual cluster size will be more than 100 and the total expected number of children across the 8 community clusters will be more than 800.*

**Response**

Thank you for taking your time to give an in-depth education on cluster sample size calculations. We followed your advice and your suggested reviews were carried out. First we dropped the calculation on design effect as suggested. Also, we inputted the estimated average number of children per cluster which currently stands at 75 in to the calculation, maintaining all other variables as suggested. This gave us a total of four clusters per study arm and a total of 8 clusters.

Considering a 10% for loss to follow-up and contamination, our final sample size stands at 660 children under five years. Thus 330 children per arm of the study. This was possible with your kind suggestion. Thank you!

**Comment**

*Secondly, your analysis section still refers to the use of paired t-tests to compare continuous outcomes - this will not be appropriate for a clustered design, since the ordinary t-test does not include adjustment for clustering. For comparing arms, please plan to use mixed effects models with random effects at the cluster level for all outcomes, but including baseline adjustment for any continuous outcomes. The analysis section also says the study is powered to detect differences in some z-scores, yet the sample size calculation is only based on a reduction in prevalence of anaemia.*

**Response**

Thank you immensely for pointing out this to us. We have modified the data analysis section, (**line 6**). Hence, for comparing arms, we would use mixed effects models with random effects at the cluster level for all outcomes, but including baseline adjustment for any continuous outcomes. Thus, we will adapt the treatment effect analysis approach considering the design effect of the study.

The aspect that referred to the following statement was expunged; *“The analysis section also says the study is powered to detect differences in some z-scores, yet the sample size calculation is only based on a reduction in prevalence of anaemia.*”

**Comment**

*Lastly, you describe stepwise variable selection for variables to be included in the adjusted models - this is not appropriate for a randomised trial. In a randomised trial you only adjust for a priori potential confounders (e.g. age and sex) and any variables used for stratification - you don't use automated processes to identify variables to adjust for because by design you don't expect characteristics to differ between the randomised groups; if you expect any differences they you stratify randomisation by those characteristics, and then you also adjust for the stratification variables. Please review these issues and if needed consult a statistician with experience in design and analysis of cluster randomised trials for further advice.*

**Response**

Thank you for pointing out this to us. The aspect of the proposed analysis describing stepwise variable selection was deleted. Multivariate data analysis would be adapted considering a priori selected confounders based on literature (age in months and sex).
