# Supplementary material for: Effects of a locally available dietary interventions counselling on the community-based management of anaemia in children under five years in Ghana: Kumbungu cluster randomized controlled trial protocol
Source: PLoS One. 2022 Apr 21;17(4):e0266157. doi: 10.1371/journal.pone.0266157 (PMC9022816; doi:10.1371/journal.pone.0266157)
Supplement: Attachment — Submitted filename: Response to reviewer comment.docx [file pone.0266157.s006.docx]

Dear Dr. Frank Wieringa

Thank you very much for agreeing to step in as new academic editor

**Academic editor comment**

Please note the results of the national micronutrient survey in Ghana, published by Wegmuller et al in 2020 in PlosOne, showing iron deficiency contributed to roughly 30% of the anemia observed in children only. Hence, in your abstract, the sentence "Most of the causes of anaemia in children under-five years are preventable." should be rephrased to 'an important part of anemia...."

**Response**

Thank you, suggestion carried out

**Reviewer 3**

**comment**

The manuscript is much improved from the previous round of reviews and I commend the authors for taking previous comments on board. I have two main points to raise in this round:

(1) the sample size calculation only works if I assume a higher ICC of around 0.035, not 0.01 as cited in point 3 on page 8, and if I ignore the coefficient of variation of 0.9 cited in point 4, which is much too high given the parameters already specified. I would recommend that the authors drop point 4 as it is determined by other parameters, and also recheck the ICC used in the calculation. Another minor point is to be clear in point 1 on page 7 that the sample size is predicated on an estimated ABSOLUTE reduction of at least 22%

**Response**

1. A higher ICC of 0.035 was used as suggested
2. Point 4 (Estimate of coefficient of variation of cluster sizes of 0.90) was dropped as suggested
3. Also, the phrase “the sample size is predicted on an estimated absolute reduction of at least 22% of the baseline prevalence”.

**comment**
(2) given that the sample size calculation is based on the prevalence of anaemia, it is incorrect to state that the primary outcome is haemoglobin (page 10). The primary outcome should be reported as anaemia, as this is what the sample size calculation is based on. The authors should then go ahead and describe how anaemia will be determined, i.e. measuring haemoglobin using an analyser and classifying children with values below [threshold] as anaemic. Most of this is already indicated in page 10-11. Further, the authors should focus the primary outcome data analysis on anaemia and not haemoglobin (haemoglobin may be analysed as a secondary outcome). If the authors intended haemoglobin to be the primary outcome, then a whole new sample size calculation appropriate for this continuous outcome is required.

**Response**

Thank you very much for this important correction. The below statement has replaced the previous phrase.

“The primary outcome measure is a reduction in the prevalence of anaemia within the study arms. The study would measure haemoglobin of children using haematological analyser and classify children with Hb <11.0 g/dL as anaemic. Since only children with Hb <11.0 g/dL would be included in the intervention, the baseline prevalence of anaemia in each arm would be 100%. At end line, the prevalence of anaemia within study arm would be determined by estimating the absolute proportion of children whose haemoglobin concentration is <11.0 g/dL.”

Thank you

Benjamin Nuertey
